# An integrative multi-omics approach points to membrane composition as a key factor in *E. coli* persistence

**DOI:** 10.1371/journal.pone.0351161

**Published:** 2026-06-29

**Authors:** Silvia J. Cañas-Duarte, Lei Sun, María Isabel Pérez-López, Cornelia Herrfurth, Lina M. Contreras, Ivo Feussner, Chad Leidy, Johan Paulsson, Diego M. Riaño-Pachón, Juan M. Pedraza, Silvia Restrepo

**Affiliations:** 1 Department of Biological Sciences, Universidad de los Andes, Bogotá, Colombia; 2 Department of Systems Biology, Harvard Medical School, Boston, Massachusetts, United States of America; 3 Albrecht-von-Haller-Institute for Plant Sciences, Department of Plant Biochemistry, University of Goettingen, Goettingen, Germany; 4 Physics Department, Universidad de los Andes, Bogotá, Colombia; 5 Laboratório de Biologia Computacional, Evolutiva e de Sistemas, Centro de Energia Nuclear na Agricultura, Universidade de São Paulo, Piracicaba, Brazil; Federal University Dutse, NIGERIA

## Abstract

Many diverse bacteria can enter non- or slow-growing states where they are transiently tolerant to antibiotics. Despite its medical importance, the genetic mechanisms underlying this ‘persistence’ remain largely unknown, especially for spontaneous (type II) persistence that arises during exponential growth. To address this challenge, here we combine genomic, transcriptomic, and lipidomic analysis to explore the persistence mechanisms. We first analyzed the genome of the high-persistence mutant *Escherichia coli* DS1 (*hipQ*) to identify candidate genes for the high-persistence phenotype. We then compared the gene expression profile of these isolated persisters to that of normally growing cells with RNA-Seq and found that the activation of stress response mechanisms is likely not important in the entrance into *hipQ*-driven spontaneous persistence during exponential growth. Transcriptomic results also suggest that modifications in the cell membrane are closely linked to persistence, as further corroborated by lipidomic profiles showing a higher level of unsaturated fatty acids in persisters compared to normally growing cells. Taken together, our results indicate that changing membrane composition is associated with persistence, and further our understanding of spontaneous persister cells from the DS1 (*hipQ*) context.

## Introduction

Clonal populations of bacteria can stochastically generate subpopulations of slow- or non-growing cells [1–4] that are transiently tolerant to multiple antibiotics. Such ‘persister’ cells have been implicated in a wide range of chronic bacterial infections [2,3]. Persister cells have also been associated with the recalcitrance of biofilms [5,6], protected from the immune system by exopolymer matrices [7]. This combination of drug-tolerance and immune-evasion prevents the complete eradication of bacterial infections. Furthermore, persisters may extend infections long enough for genetic resistances to emerge [7,8]. The persistence phenomena are widespread among pathogens, including *Staphylococcus aureus*, *Pseudomonas aeruginosa*, and *Mycobacterium tuberculosis* [2,9,10], and therefore of high public health priority.

From an ecological perspective, creating small subpopulations that survive otherwise bactericidal treatments may serve as an adaptive, bet-hedging strategy against catastrophic events [1,11] for the population as a whole. In support of this hypothesis, some Toxin-Antitoxin (TA) loci such as *hipAB* and *istR/tisB*, and stress response mechanisms have both been implicated in the generation of persister cells [12–20]. Another proposed pathway for induction of persistence is through TolC efflux pumps [21], which also display great heterogeneity across populations [22]. These findings illustrate both how external signals can influence the transition to persistence and the variety of factors that determine the state. They also have in common an important connection with the membrane. Despite these advances, the specific mechanisms of multitolerance remain largely unknown [22,23]. The variety of insults that they can survive includes not only different types of antibiotics, but also alkaline and enzymatic lysis [24]. Furthermore, dormancy alone is not necessary or sufficient to provide persistence [25]. This indicates that there is still much to be discovered about the persistence state itself and how it results in multitolerance.

**Fig 1:**
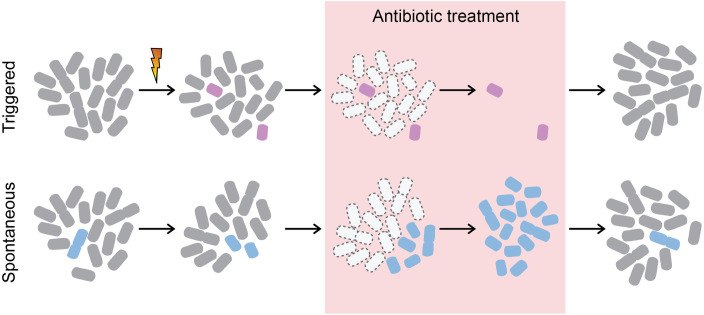
Schematic of the differences between triggered and spontaneous persisters. Triggered persisters are generated in bacterial populations upon the occurrence of a trigger event (i.e., starvation, antibiotics, acid stress, etc.) and are characteristically non-growing while in the persister state. Spontaneous persisters stochastically appear in bacterial populations. In DS1, spontaneous persisters exhibit slow growth in the presence of antibiotics [1].

Persisters have been phenotypically classified as triggered (type I) or spontaneous (type II), depending on whether they are directly caused by stress or arise in growing populations without any known external trigger. These two persister types indeed correspond to distinctly different cell states in *E. coli* [1,4] and seemingly involve different sets of mechanisms (Fig 1). However, either type has been hard to study because the persister state is extremely rare. Many studies have therefore relied on specific mutants that increase the persister frequency, hoping that they do not distort their wild-type counterpart in other ways. Specifically, for triggered persisters, mutant strain *hipA7* (TH1269) [1,4,17,26,27] has successfully helped to identify the involvement of TA modules and stress response mechanisms [2,8,17,23,28].

For spontaneous persisters, the high-persistence strain *E. coli* DS1 was isolated via mutagenesis of the parent *E.* *coli* strain KL16 [1,4,29]. By first using conjugative mapping and later transducing wild-type genome fragments via P1vir into DS1 to check for the loss of high-persistence, it was found that a *hipQ* locus near the *leu* operon at the 2 min position of the DS1 chromosome was necessary for the high-persistence phenotype of this strain [27]. However, attempts to transduce the *hipQ* phenotype to a wild-type background failed, which suggests this locus is not sufficient and that additional unidentified mutations present in DS1 genome are required [27]. Using microfluidics and microscopy, spontaneous persisters from the DS1 strain were later visualized as slow-growing cells (~160 + /- 30 min doubling-time) that arise spontaneously from an exponentially growing bacterial population [1,24]. Entrance into this slow-growth state seems independent of antibiotics and cells showed division both before, during and after a transient antibiotic treatment [1,24]. A recent study [30] presented a mutation in the *ydcI* gene (at 32 min chromosomal location) as solely responsible for the *hipQ* persister phenotype. Interestingly, these two studies contradict each other both in the gene location and number of mutations required. In addition, due to the higher difficulty in isolating spontaneous persisters [24], their overall persistence mechanism remains largely unknown.

Using our previously-published protocol capable of selectively enriching triggered versus spontaneous persisters from bacterial populations [24], we performed comprehensive genomic, transcriptomic and lipidomic analyses on *hipQ* exponential-phase persisters. Genomically, we identified 59 novel missense and nonsense SNP mutations in the DS1 genome that we propose as candidates responsible for its high spontaneous persistence phenotype. Notably, we found that *ydcI* has no mutation in this strain. Furthermore, there is no DS1 strain-specific mutation near the *leu* operon where the *hipQ*-locus was proposed to be located [27]. By assessing the persister frequencies of the relevant mutants, we show that the mutations responsible for the *hipQ* phenotype of the DS1 have not yet been identified, despite previous reports. Transcriptomically, we tested whether the previously reported genetic mechanisms of persistence induction, such as stress response mechanisms, are also involved in the generation of exponential phase persister cells from the strain *E. coli* DS1 (*hipQ*) [2,23]. Additionally, the transcriptomic analysis of these persister cells showed no evidence of SOS response activation, contrary to several reports [12,13,16,23]. Finally, our lipidomic data suggests that modifications in the physicochemical properties of the cell membrane could be related to the formation of persister cells and contribute to their multitolerance to bactericidal agents. We believe these results not only broadly add to the characterization of the persister states, but also serve as a springboard for future mechanistic analyses.

## Results

### Novel SNPs identified in *E. coli* DS1 genome

**Fig 2:**
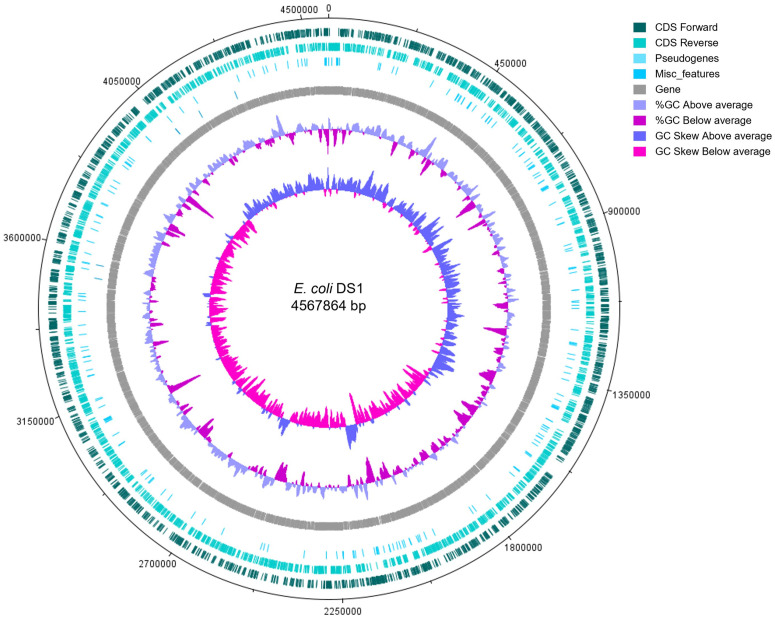
Circular representation of the novel genome assembly and annotation of *E. coli* DS1. Circular plot of the annotated DS1 genome was created using DNAplotter [36]. The tracks from the outside represent: (1) CDS forward; (2) CDS reverse; (3) pseudogenes; (4) miscellaneous features; (5) genes; (6) %GC plot; (7) GC skew [(GC)/(G + C)]. For the %GC and GC skew plots, a window size of 10,000 bp and a step size of 200 bp were used.

To identify the genetic mechanisms related to the high spontaneous persistence phenotype of *E. coli* DS1, we first sequenced the DS1 genome, obtaining 2.5 million clean reads with a minimum length of 60 bp. The average coverage in the *de novo* genome assembly was 82.85 × , with a maximum coverage of 358 × , estimated using Tablet [31]. Following the *de novo* assembly, we obtained a single pseudo-molecule of 4,567,805 bp with 47 gaps and 531 N’s (Table 1). After the annotation step, 4,554 genes were predicted (Fig 2).

**Table 1:** Metrics of the *E. coli* DS1 genome assembly.

Total number of scaffolds	1
Sum (bp)	4,567,805
Total number of N’s	531
Number of GAP’s	47
Sum (bp) no N’s	4,567,274

Assembly of the DS1 genome was performed *de novo* using CLC, obtaining initially 104 contigs. The scaffolding process was performed using first SSPACE [32] with clean PE reads from the sequencing of both the genome and transcriptome to obtain 58 scaffolds. The genome of *E. coli* MG1655 was used to refine the assembly using the PAGIT pipeline [33]. Finally, Gapfiller [34] was used to close gaps. The RAST [35] annotation server was used to predict and annotate genes in the DS1 genome.

The *E. coli* DS1 strain was evolved from the KL16 [37] parent strain. To our knowledge, the only publicly available KL16 genome sequence is that of KLY, namely KL16 with an integrated YFP segmentation marker (GenBank: CP008801.1). The comparison of KLY, MG1655 and DH10B to reference genomes revealed a total of 153, 255 and 349 SNPs (including small indels), respectively (Table 2). As none of the reference strains show a high persistence phenotype, we only considered the 112 novel SNPs that were detected against all three reference genomes for further analyses (S1 File). The genomic analysis performed also showed the presence of the F plasmid integrated into the chromosome [27,29]. No large insertions/deletions or genomic arrangements were detected with respect to the KLY genome.

**Table 2:** Analysis of Single Nucleotide Polymorphisms (SNPs) detected in the genome of *E. coli* DS1.

Type	Strain	Count	Percent/ (Total SNPs)
MISSENSE	MG1655	132	43.40%
	KLY	58	37.90%
	DH10B	122	73.05%
NONSENSE	MG1655	3	1.57%
	KLY	1	0.65%
	DH10B	3	1.80%
SILENT	MG1655	109	52.60%
	KLY	89	58.17%
	DH10B	224	64.20%

The genomes of the KLY (KL16 with YFP) parental strain, the wild-type strain MG1655, and the DH10B strain were used as references; we detected a total of 153, 255, and 349 SNPs, respectively.

As its parent strain KL16, *E. coli* DS1 encodes the *relA1* and *spoT1* mutations, along with several other polymorphisms when compared to the MG1655 strain. It has been previously reported that strains with *relA1* and *spoT1* mutations have deficient stringent response [38]. The wild-type persistence levels of the KL16 strain and the fact that the *hipQ* phenotype of DS1 is most relevant during exponential phase and conditions free of external stressors, strongly indicate that these mutations are not directly involved in the *hipQ* phenotype.

Some of the identified polymorphisms appear in genes whose functions were previously reported to be related to persistence, such as stress response mechanisms, DNA replication, and catabolic processes of amino acids and carbon sources [8,17]. We found a novel mutation in the *hipA* locus (A242V) different from the previously characterized *hipA7* mutation known to confer a high persistence phenotype [17]. However, this mutation, although not generally considered a conservative change, does not appear to generate any significant conformational change in the protein (S2 File), although it is located near the active site of this kinase. We also identified various mutations in genes involved in the metabolism and transport of lipids such as *fadD, ytfN (tamA)* and *ytfM* (*tamB*); cell wall homeostasis such as murB, *yceG* (*mltG*) and *amiB*; and transmembrane transport such as *ompF and ompN*. As discussed in the next section, these results suggest the existence of modifications in cell envelope physicochemical properties in persister cells, as several components related to the cell envelope were found to be overrepresented amongst the set of genes in which novel mutations were identified for DS1 (Table 3). As noted above, our genomic analysis found no mutations in the gene *ydcI*, recently reported as the gene responsible for the high spontaneous persistence phenotype [30].

**Table 3:** Overrepresentation analysis of the cellular components represented in the set of genes with novel SNPs in DS1.

Go term (Cell component)	Count	p-value	Fold enrichment	Fisher Exact test
Cell envelope	18	1.1E-03	2.3	4.3E-04
Outer membrane-bounded periplasmic space	11	1.8E-02	2.3	7.5E-03
Intracellular organelle lumen	3	2.5E-02	11.7	1.8E-03
Organelle lumen	3	2.5E-02	11.7	1.8E-03
Membrane-enclosed lumen	3	2.5E-02	11.7	1.8E-03
Membrane	45	3.0E-02	1.2	2.3E-02
Periplasmic space	12	3.2E-02	2	1.5E-02
Intracellular membrane-bounded organelle	4	4.2E-02	5	7.5E-03
Membrane-bounded organelle	4	4.5E-02	4.9	8.3E-03
2-iminoacetate synthase complex	2	4.6E-02	42.8	5.4E-04
TAM protein secretion complex	2	4.6E-02	42.8	5.4E-04
Pore complex	3	7.0E-02	6.8	9.1E-03
Cell outer membrane	7	8.2E-02	2.3	3.3E-02
Outer membrane	7	9.9E-02	2.2	4.2E-02

To analyze which cellular components were significantly overrepresented amongst the set of mutated genes in DS1, PANTHER [39,40] was used to map the genes to UniProt accession numbers. DAVID [41,42] was then used to test for overrepresentation of cellular components, using as reference the complete genome of *E. coli* K-12.

**Fig 3:**
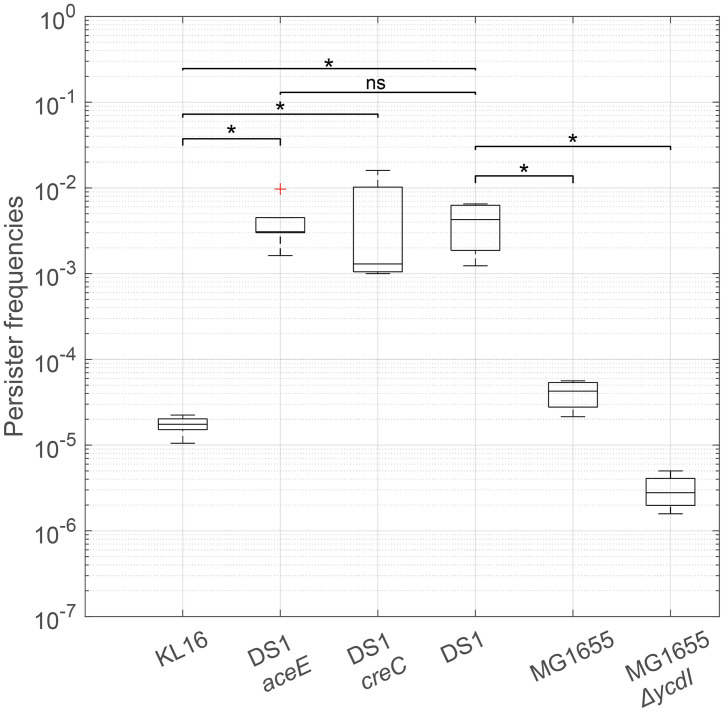
Comparison of persister frequencies of previously reported *hipQ* related mutations in the wild-type strains (KL16 and MG1655) and the *hipQ* (DS1) strain. Persister frequencies were determined by ampicillin treatment for 3 hours, as described previously [1]. Ampicillin (100 µg/mL) was added to the growing culture when it reached an OD600 = 0.4. Time 0 (before addition of antibiotic) and t = 3 hours were serially diluted and plated on LB agar to determine CFUs. Values represent the average of 3 biological replicates. Two-sample t-tests were performed to determine if the differences between the persister frequencies of the indicated pairs of strains are significant (p-value < 0.05).

To further corroborate this, we designed specific primers for the *ydcI* gene and performed Sanger sequencing. Again, no mutations were detected. Furthermore, we tested the persister frequency to ampicillin of a Δ*ydcI* strain generated by P1 transduction from the KEIO collection [43] into MG1655. According to the previous report [30], the MG1655 Δ*ydcI* strain should show a similarly high persister frequency as that of the DS1. Our results, however, show this is not the case, with this strain having persister levels close to that of the MG1655 wild-type (Fig 3 and S11 File). In addition, we searched for mutations located within ~100 kb from the *leu* operon, the proposed site of the *hipQ* locus [27]. To our surprise, DS1 has no strain-specific point mutations in the said region. Instead, it contains two SNPs inherited from its KL16 parent strain: *creC* R77P and *aceE* A20T. To assess whether these two mutations contribute to high-persistence, we restored each of these mutations independently with P1 transduction using the respective wild-type gene from the MG1655 strain and moved it into the DS1 strain and then tested the persister frequency to ampicillin. For both the DS1 *creC* and DS1 *aceE* restored strains, we found no significant changes in their persister frequencies when compared to the original DS1 strain, indicating that these mutations inherited from KL16 have no effect on the hipQ phenotype (Fig 3 and S11 File). So far, our results suggest that all previous findings on the *hipQ* genetics are likely incorrect. Therefore, we conclude that the genetic mechanism of *hipQ* is not yet determined and remains an open question.

### Differentially expressed genes in DS1 persisters were distinct from previously reported transcriptomes of persister cells

Due to the large number of SNPs detected, pinpointing the mechanism with genome alone is challenging, especially since multiple mutations are likely involved [27]. Therefore, we carried out transcriptomic analysis. Previously, persisters have been routinely isolated by antibiotic treatments that last a few hours [1,8,10,16]. Alternatively, triggered persisters have been enriched using flow cytometry with fluorescent reporters of ribosomal genes like *rrnbP1* [44]. Depending on the choice of antibiotics, cell physiology could be differentially affected during persister isolation [16,45]. Since spontaneous persisters emerge independently of antibiotic treatment, and to avoid antibiotic-induced biases, we used a previously published lysis protocol that isolates persisters in under 30 min [24] (S3 File). To reduce the probability of carry-over triggered persisters from the stationary phase starting culture, a sufficiently large dilution was performed before persister isolation in early exponential phase [4]. Critically, persisters enriched in DS1 during exponential phase maintained both their viability and characteristic slow growth dynamics for DS1 spontaneous persisters (S4 File) [24]. As shown previously using time-lapse microscopy, the fraction of isolated cells that don’t exhibit growth within a 2-hour window is negligible [24]. As this key phenotype is preserved, the associated experimental alterations to the cellular components are likely minimal. From the isolated DS1 persisters, we performed transcriptomic analysis and compared it to the transcriptome of exponentially growing, regular cells of DS1.

Utilizing the paired-end and strand-specific reads obtained during the RNA-Seq analysis, we assembled a complete gene catalog of expressed genes of *E. coli* DS1 persister cells and exponential phase cells using 27,525,259 and 40,130,287 paired-end reads, respectively, with an average length of 78 bp. For DS1 persister cells, we assembled and annotated 5,768 transcripts; 5,748 of these transcripts displayed BLAST hits, whereas the number of transcripts was 4,393 for the exponentially growing cells.

A total of 301 statistically significant differentially expressed genes (DEG) were detected. Of these, 217 were found to be down-regulated and the remaining 84 up-regulated. In the list of up-regulated genes, we noticed the overall absence of genes related to stress response mechanisms that were previously reported to be involved in the generation of persister cells [8,22,28]. Notably, genes participating in the SOS mechanism did not display differential regulation. Several genes related to other stress-response mechanisms were found in the list of down-regulated genes (S5 File).

Seven genes involved in lipid metabolism and modifications were found to be differentially regulated, such as FadB, along with genes involved in the regulation of other cell envelope components such peptidoglycan and LPS. This is consistent with the identification of 9 novel mutations in genes related to lipid pathways in the DS1 genome. 16 genes from TA modules were found to be differentially expressed during this type of persistence. But their behavior varied across TA modules: some were down-regulated, such as, *hipB, dinJ* and *relB,* whereas others, such as *pspB*, were up-regulated. In particular, we found that the *tisB* toxin was down-regulated.

### Lipid metabolism is associated with DS1- persistence

We analyzed genes that were differentially expressed to detect whether some biological functions were significantly over- or underrepresented in this cluster. We found that genes related to regulation of translation, regulation of protein metabolic processes, several macromolecules biosynthetic processes, response to stimuli and response to stress were significantly overrepresented in the differentially expressed gene cluster, highlighting the importance of the regulation of these functions in DS1 exponential-phase persister cells.

**Fig 4:**
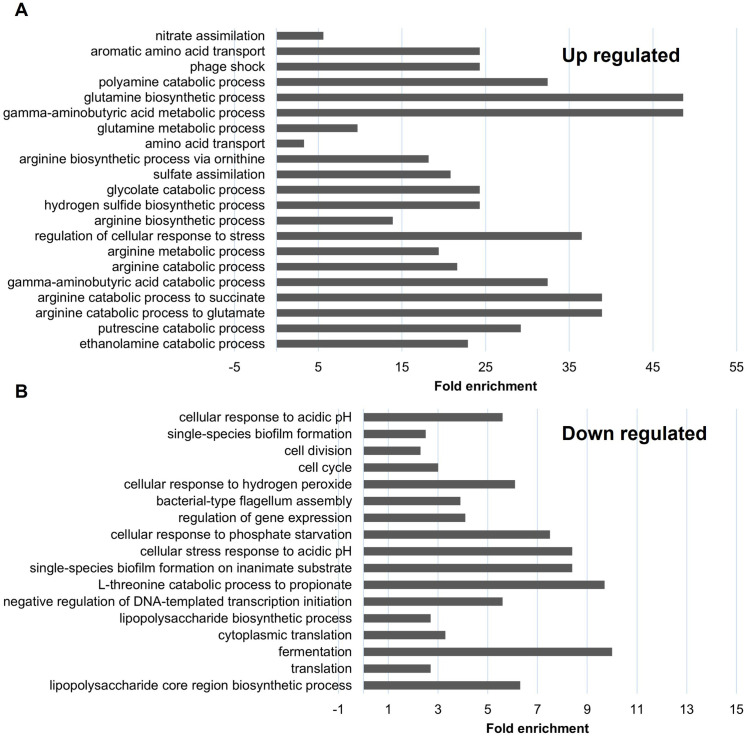
Biological processes significantly overrepresented during DS1 persistence. To analyze which biological processes were significantly regulated during spontaneous persistence in DS1, Blast2GO [46] was used to map all the genes from the subsets of upregulated and downregulated DEGs. DAVID [41,42] was then used to test for overrepresentation of biological functions in the subsets of (A) up-regulated and (B) down-regulated. As noted, several processes involving both DNA and RNA binding and metabolism of proteins are overrepresented in the set of down-regulated genes in DS1 persister cells, whereas the metabolism of cell envelope components is overall overrepresented in both the up-regulated and the down-regulated clusters, indicating strong regulation of this function during spontaneous persistence. Only biological functions found to be overrepresented with a Bonferroni score < 0.005 are shown and an FDR < 0.0005.

We separately analyzed the clusters of up-regulated and down-regulated genes to identify the biological functions and processes related to spontaneous persistence in *E. coli* DS1. In the down-regulated gene cluster during DS1 exponential-phase persistence, we found that genes related to cell division, responses to stimulus and stressful conditions, synthesis of macromolecules, translation and overall homeostasis-related processes were significantly overrepresented (Fig 4B).

When analyzing the cluster of up-regulated genes, we found that functions related to the intake of carbon sources, polyamine metabolism, and general catabolic processes were overrepresented. Biological functions related to lipid metabolic processes were significantly overrepresented (i.e., ethanolamine catabolic process), suggesting that modifications in the cell membrane composition and physicochemical properties are important during spontaneous persistence (Fig 4A).

### RT-qPCR validates all tested differentially expressed genes predicted by our RNA-Seq analysis

We selected a subset of 12 genes (S6 File) out of 301 total genes found to be differentially expressed with the RNA-Seq analysis for validation: 4 and 8 from the up- and down-regulated cluster, respectively. Besides the differential expression, the selection criteria for this subset of genes included biological functions and their relationship with mechanisms previously related to the persistence phenomenon [8,22,28].

To select appropriate reference genes for the RT-qPCR experiments, the expression profile of previously reported housekeeping genes in the RNA-Seq dataset was analyzed. The expression of the genes *opgH* and *dxs* appeared to be suitable as RT-qPCR reference genes, whereas other commonly used housekeeping genes in *E. coli*, such as *recA* and *mdh*, showed variations in their abundances when comparing the *E. coli* DS1 strain during its spontaneous physiological state to its exponential growth.

All tested genes displayed similar expression profiles in the qPCR analysis, as reported by our RNA-Seq analysis (S7 File, including the housekeeping genes *opgH* and *dxs*).

### Identification of genes with non-synonymous mutations related to gene expression changes in *E. coli* DS1 persisters potentially responsible for its high persistence phenotype

Firstly, we identified 19 SNPs directly related to the differential expression of genes during persistence (S8 File). Amongst these differentially expressed genes a non-synonymous mutation in *narZ*, which encodes for the nitrate reductase Z subunit α, is related with the up-regulation of the *narZWY* operon. Significantly, we found that a non-synonymous mutation in the transcriptional terminator Rho correlates with the differential expression of at least 16 genes from the putative Rho-dependent termination regulon [47]. The remaining 17 mutations identified exhibit correlations between regulators like SoxR and the mutated genes.

We then decided to directly assess the transcriptomic expression profiles of the genes found to be mutated in the DS1 genome. For this, we started by focusing on the cluster of 57 unique genes in which at least one non-synonymous SNP was detected. From this cluster, we identified 28 genes with a log2 Fold change >+/- 1 in gene expression from the differential expression analysis of DS1 exponential-phase persisters compared to non-persisters (Table 4). We then analyzed each of the mutated genes in terms of biological function, and assessed their possible relevance to persistence by combining their gene expression changes in persister cells with several reported factors involved in persistence such as stress response mechanisms, DNA replication and cell division and catabolic processes of amino acids and carbon sources [8,17,28]. With this functional analysis, we selected 10 additional genes carrying non-synonymous mutations that we consider might be potentially involved in *hipQ*-high persistence but that do not exhibit large expression changes (Table 4), whose functions we discuss in the Discussion section below.

**Table 4:** *E. coli* DS1 genes with novel non-synonymous mutations potentially involved in *hipQ*-driven persistence.

Gene	SNP Effect	Log2FC	Effect	Group	Function
*rhsA*	Truncation	−2.90	Down	1	Hydrophilic protein of unknown function [48]
*ydiM*	Substitution	2.21	Up	1	Multidrug transporter [49]
*paaD*	Substitution	1.83	Up	1	Predicted subunit of a phenylacetate-CoA oxygenase [50]
*ydbD*	Substitution	1.74	Up	1	Possibly involved in detoxification of methylglyoxal (MG) [51]
*ompN*	Substitution	1.72	Up	1	Outer membrane porin [52]
*orn*	Substitution	−1.67	Down	1	Oligoribonuclease [53]
*treB*	Substitution	−1.62	Down	1	Trehalose-specific PTS enzyme IIBC component [54]
*narZ*	Substitution	1.61	Up	1	α subunit of nitrate reductase Z [54]
*ynbC*	Substitution	1.48	Up	1	Putative hydrolase/methyltransferase [55]
*yncE*	Substitution	−1.41	Down	1	Possibly involved in iron uptake [56]
*yeaI*	Substitution	1.41	Up	1	c-di-GMP binding protein CdgI [57]
*citG*	Substitution	1.40	Up	1	Triphosphoribosyl-dephospho-CoA synthase [58]
*ydhV*	Substitution	1.32	Up	1	Putative oxidoreductase [59]
*mgtA*	Substitution	1.26	Up	1	Mg2 + importing P-type ATPase [60]
*flhA*	Substitution	−1.15	Down	1	Component of the flagellar export apparatus [61]
*yjhC*	Substitution	−1.06	Down	1	KpLE2 phage-like element NanY [62]
*yjaA*	Substitution	−1.05	Down	1	Involved in the cellular response to hydrogen peroxide and acid stress [63]
*rhsD*	Substitution	1.03	Up	1	Hydrophilic protein of unknown function [48]
*yceG*	Substitution	0.91	Up	2	Endolytic murein transglycosylase MltG [64]
*katE*	Substitution	0.89	Up	2	Monofunctional catalase HPII [65]
*hflX*	Substitution	−0.73	Down	2	Ribosome rescue factor HflX [66]
*pgi*	Substitution	−0.69	Down	2	Glucose-6-phosphate isomerase [67]
*hipA*	Substitution	−0.66	Down	2	Serine/threonine-protein kinase, TA module [26]
*rsxG*	Substitution	−0.51	Down	2	SoxR [2Fe-2S] reducing system protein RsxG [68]
*rho*	Substitution	−0.34	Down	2	Transcription termination factor Rho [69]
*ompF*	Frame Shift	−0.04	Down	2	Outer membrane porin [70]
*rseB*	Substitution	−0.02	Down	2	Anti-sigmaE factor [71]
*rpoB*	Substitution	−0.01	Down	2	RNA polymerase subunit β [72,73]

18 genes were selected with changes in expression > 2-fold in persisters compared to the exponentially growing cells from which they emerged (Group 1); 10 additional genes were selected based on their biological function having been linked to persistence but with smaller or undetected changes in expression (Group 2).

### Analysis of fatty acid profiles validates the occurrence of significant membrane modifications in persister cells

The results of our RNA-Seq analysis of DS1 persisters isolated during exponential growth show that genes involved in lipid metabolism, normally involved in the modulation of fatty acid chemical structure, become differentially expressed, indicating a possible role of lipid composition of the membrane during persistence.

**Fig 5:**
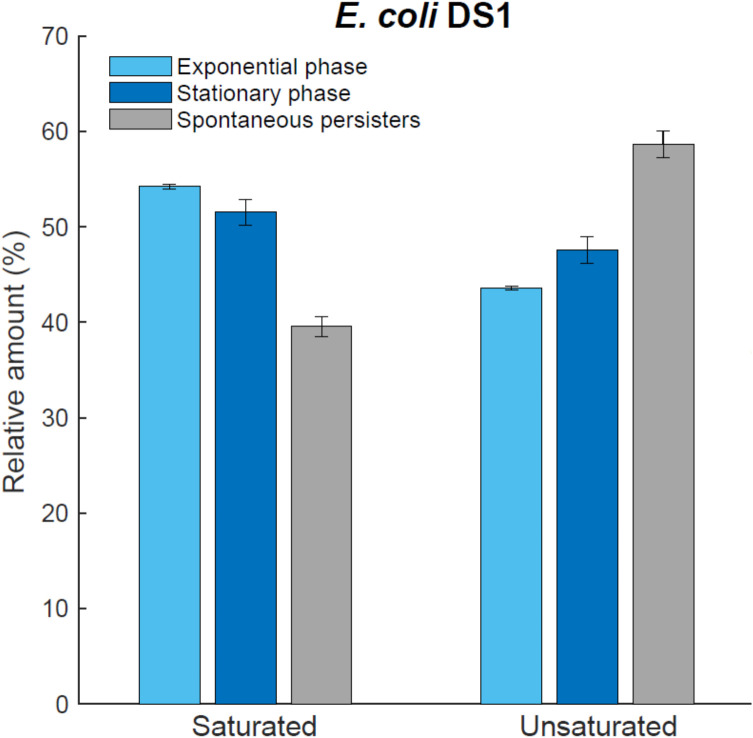
Fatty acid chain profiles evidence the occurrence of membrane modifications in persisters. The lipid composition of persister cells is markedly different from that of the cells in the physiological state in which they are generated. The membranes of DS1 persisters, generated during exponential growth, are highly enriched in unsaturated lipids, consistent with an overall increase in the fluidity (decrease in lipid packing) of the cell membrane in persister cells, compared with exponentially and stationary phase cells.

**Fig 6:**
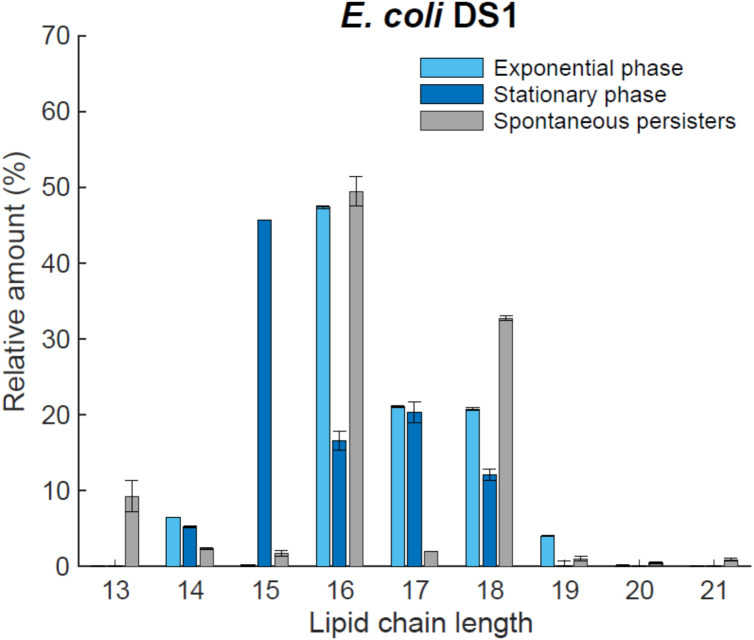
The fatty acid chain-length profiles of persisters are distinct from normally growing cells. DS1 persisters isolated during exponential growth are enriched in 18 carbon chains compared with cells in exponential growth, which is consistent with an increase in the unsaturated 18:1Δ9 species. The average chain does not exhibit a significant change compared to normally growing cells.

Overall, significant differences were found when comparing the fatty acid profiles of persister cells with non-persister cells during both the exponential growth and stationary phases (Fig 5 and S9 File). In contrast to the triggered persisters, spontaneous or Type-II persisters in DS1 have been shown to be dividing, albeit with a prolonged division time of ~160 ± 30 min [1,24]. Whether spontaneous persisters in other strains or species exhibit slow growth, complete arrest, or other behavior remains an open question. Interestingly, persisters isolated from DS1 during exponential growth increase the proportion of unsaturated fatty acids in their membrane, from an average of 41% in non-persisters to more than 58% in persisters, suggesting an overall increase in membrane fluidity in spontaneous persistence. No changes in the average fatty acid chain length were observed (Fig 6)

## Discussion

Despite a great and long-standing interest in bacterial persistence, much is still unknown about the underlying biochemical and molecular mechanisms by which they are generated as persister cells are too infrequent and their transient nature makes them difficult to study. Recent consensus has categorized persisters largely into two types: triggered and spontaneous, previously known as type I and type II [4]. Persistence could also arise from other mechanisms such as balanced division and deaths under antibiotic treatment [74]. Furthermore, host tissues could cause phenotypic variations of bacterial pathogens and contribute to persistence [75].

For induced persisters, a great deal has been learned from the high persistence strain *hipA7*, mutated in the gene for the HipA toxin. For spontaneous persisters, *hipQ* has played a similar role in phenotypic analyses, as the only published mutant to our knowledge that substantially increased spontaneous persistence frequency. However, as opposed to *hipA*, the *hipQ* designation does not refer to any identified gene. A major early attempt using conjugative mapping [27] failed to identify specific genes, and though a recent paper suggested a gene [30], that polymorphism does not appear to exist in the DS1 *hipQ* strain. We here present a multi-omic approach of the classic *hipQ* persister strain, and for the first time identify not only key polymorphisms, but also expression profiles and lipid modifications.

Our analysis of the complete *E. coli* DS1 genome identified 121 polymorphisms unique to this strain, including a cluster of 57 genes with non-synonymous mutations potentially involved in the generation of persister cells. One notable mutation found in the DS1 genome is a novel polymorphism in the coding sequence of the HipA toxin gene, a single point mutation changing Alanine for Valine (A242V). This mutation is distinct from the HipA7 mutation (G22S+D291A) responsible for the hip phenotype of *hipA7* strain [17,26,76]. However, we do not expect the A242V mutation to cause significant conformational changes to the HipA protein, since alanine and valine are structurally and functionally very similar. Several mutations occurred in genes previously implicated in persistence in *E. coli* cells, such as genes related to stress response mechanisms, biofilm formation, quorum sensing and catabolic processes [6,8,10,13,17,28,77,78]. Notably, the analysis of the DS1 genome supported the hypothesis that modifications to the lipids composing the cell membrane might influence multitolerance.

We further sequenced the complete transcriptome of *E. coli* DS1 exponential-phase persisters using exponentially growing cells as a control. A total of 180 genes were differentially expressed: 64% were down-regulated, and 36% were up-regulated. Many genes related to carbohydrate metabolism were up-regulated, whereas genes encoding for cell division, protein synthesis and cell homeostasis processes were down-regulated. This regulation contradicts the idea that the mechanism of multitolerance is primarily because of metabolic inactivity [2,23]. Genes in Toxin-Antitoxin modules were also differentially regulated during persistence, but some were upregulated while others were down-regulated. For example, the TisB toxin was down-regulated, despite the fact that it has been shown that TisB can play a role in antibiotic-induced persistence [2,13].

Several genes related to stress response mechanisms were down-regulated, whereas only two stress-related genes, *zraP* and *pspD*, were up-regulated. This regulation was contrary to previous studies on triggered persistence [2,8,23,28]. Notably, all genes related to the SOS response showed no transcriptomic change in expression or were beyond our resolution power. This particular and striking difference between the current expression profile of DS1 persisters and all previous transcriptomic studies performed on persisters could be caused by the inherent differences between both persister types. Notably, previous studies routinely isolate persisters by using antibiotics, e.g., fluoroquinolones that induce the SOS response. The induction of the SOS response by antibiotics during the isolation of persister cells in all previous studies is likely to have caused significant alterations to the expression profiles of the isolated persisters. Similarly, the activation of the stringent response, through the accumulation of the alarmone guanosine tetra/penta-phosphate ((p)ppGpp) has been recently reported to play a role in the formation of all persisters types in the Gram-positive *Bacillus subtilis* [79]. Despite taking great care to deplete starvation-induced persisters for their spontaneous persister assays, the antibiotic treatments for isolation could have led to transcriptional changes or antibiotic induced persister formation in these assays. Further, as no measurements were made of the growth dynamics of the isolated *Bacillus* persisters, in order to verify if they have the characteristic slow-growth [1] of spontaneous persisters, it is not possible to know if the role of (p)ppGpp extends to spontaneous persistence in general. Our findings suggest that the induction of stress mechanisms, such as the stringent response and oxidative stress, only become a significant trigger to induce persistence formation if the SOS response has been previously induced, as suggested by the absence of these mechanisms in the set of upregulated genes in DS1 persisters. Importantly, we, however, do not discount the possibility that global regulators like RpoS or (p)ppGpp could have a significant role in the ability of spontaneous persisters to survive antibiotics, downstream of the entrance into this state.

Gene expression information could significantly aid in the discovery of the genes responsible for the high persistence phenotype of DS1. However, expression patterns can be hard to interpret, as they might be causative, responsive, or independent of persistence. But by correlating the discovered SNPs in the genome of *E. coli* DS1 with transcriptome data, we propose 28 candidate genes that may be involved somehow in persistence (Table 4). For example, the outer membrane porin *ompF*, which is known to mediate the entry of various antibiotics [80,81] was found to have suffered a deletion which resulted in a predicted frame shift, which might severely affect the functionality of this porin. Another interesting candidate mutation was found in the MltG protein (formerly *yceG*). MltG is an inner membrane enzyme with endolytic murein transglycosylase activity which has been proposed to terminate nascent peptidoglycan synthesis [64] and its deletion has been reported to reduce sensitivity to ampicillin [82]. Additionally, the non-synonymous mutations found in the genes *rho, rpoB, rseB, hflX* and *rsxG* could broadly affect gene expression via alteration of transcription factor activity, translation regulation and mRNA stability, which could also increase noise in gene expression favoring the entrance into alternate phenotypic states.

A notable result of our transcriptomics analysis was the overrepresentation of genes related to fatty acid metabolism and lipid modifications among the biological functions strongly up-regulated among DS1 persisters. These results not only correlate with some of the mutations found in the DS1 genome but also support previous findings about differences in the cell membrane of persister cells [24]. The chemical structure of fatty acids determines the level of lipid packing and influences the biophysical properties of bacterial membranes [83]. We therefore analyzed the fatty acid composition of persister cells and compared the composition with normally growing cells. The fatty acid composition of persisters differed significantly from that of the normal cells. Specifically, persisters isolated during exponential growth phase appear to increase their membrane fluidity. However, it is important to note that although the lytic protocol used to isolated persisters in this study acts in < 15 minutes and has been shown to reproduce both the published persister frequencies and growth phenotypes in both wild type *E. coli* and in the *hip* (*hipA7* and *hipQ*) strains [24], we cannot fully discount the possibility that this method might add a selectivity for cells with modified membrane properties or for other cell types that are non-persisters, such as viable but non-culturable (VBNC) cells.

The regulation of the physicochemical properties of the cell membrane is a known bacterial strategy to survive challenging environments [83]. Previous studies have also shown a relationship between membrane fluidity and tolerance to certain antibiotic agents [84]. Therefore, alterations in membrane composition and fluidity emerge as strongly associated with the multitolerant state.

## Conclusions

In summary, we combined multi-omics methods to characterize the high persistence strain DS1 *hipQ* in terms of polymorphisms as well as broad changes in the transcriptome and lipid composition. This revealed many striking changes, excluded previously proposed genes and locus claimed to cause persistence, and identified a set of new candidate genes. Finally, we noted differences in the membrane lipid composition between persister and non-persister cells, thus opening a new avenue of research on the biochemical mechanism of multitolerance.

## Methods

**Bacterial strains and growth conditions.**

The bacterial strains used in this work and their relevant characteristics are described in Table 5. Bacterial cultures were grown in Luria-Bertani (LB) medium at 37°C and 200 rpm unless otherwise specified. For exponential growth, samples were taken at OD600 = 0.4. The morphological characteristics of strains DS1, KL16 and MG1655 were analyzed at this point and are depicted in S10 File.

**Table 5:** *Escherichia coli* strains used in this study.

Strains	Relevant features	Source or reference
TH1268	MG1655 zde264::Tn10	Korch et al, 2003
TH1269	MG1655 *hipA7* zde264::Tn10	Korch et al, 2003
DS1	hipQ	Wolfson et al, 1990
DSY	hipQ with yfp-Cam cassette	Fridman et al., 2014
KL16	K-12 *λ-, e14-, relA1, spoT1, thiE1*	Low B., 1968
MG1655	MG1655 6300	
MG1655 ΔydcI	MG1655 6300 *ΔydcI*	This study

***Escherichia coli* DS1 genome sequencing, assembly and annotation.**

Total DNA was purified from a stationary phase culture of *E. coli* DS1 using the GenElute Bacterial Genome Extraction Kit (Sigma-Aldrich, St. Louis, USA) according to the manufacturer’s protocols. The DS1 genome was sequenced on an Illumina HiSeq 2000 instrument using the 2 × 90 paired-end technology with a 500 bp insert size at BGI Genomics (Shenzhen, China).

FastQC (Babraham Bioinformatics, Cambridge, United Kingdom) was utilized to visually inspect quality metrics of the raw reads. Reads were clipped, quality trimmed and quality filtered (with a minimum read length of 60 bps and a quality threshold of 20) using Flexbar [85].

Clean reads were then *de novo* assembled using the CLC Assembly Cell (CLC Bio, Aarhus, Denmark). PET scaffolding was performed using the SSAKE-based Scaffolding of Pre-Assembled Contigs after Extension (SSPACE) v2.0 [34]. The PAGIT (Post Assembly Genome Improvement Toolkit) [33] was utilized for reference-guided contig extension using ABACAS [86], PET gap closing was performed using IMAGE [87] and the quality assessment of the assembly was made using iCORN [88]. Finally, Gapfiller was used [34] to close the majority of the remaining gaps. DS1 genome annotation was performed using the Rapid Annotations using Subsystems Technology program [35]. For visualization, a circular representation of the DS1 genome was created using DNAplotter [36].

### Whole genome SNP detection

SNP calling was performed as described by the BROAD GATK Best practices guidelines (v3) using both *E. coli* MG1655 and DH10B genomes as references. Briefly, the sequenced small reads were mapped against each reference genome using the Burrows-Wheeler Aligner (BWA) [89], and the coverage depth was analyzed using the Genome Analysis Toolkit (GATK) [90], obtaining a 94 × coverage for each genome. Next, duplicates were marked using Picard [http://picard.sourceforge.net] before performing local re-alignments. For the base quality recalibration step, we built a database of polymorphic sites in *E. coli* using the genomes of strains BL21, S88, 0127:H6 E2348/69, O42, ETEC H10407, DH10B and MG1655, employing progressive Mauve [91] for the multiple sequences alignment. Finally, SNPs and small indels in the genome of *E. coli* DS1 were called against each reference genome with GATK [92].

### Analysis of overrepresented GO terms in the set of genes encoding novel mutations in *E. coli* DS1

The set of genes identified to have novel mutations, with respect to the three reference strains, was singularly mapped to UniProt accession identifiers using PANTHER [39,40]. The 95 unique entries were then analyzed for overrepresentation of Cellular component GO terms using DAVID 6.7 [41,42] with Fisher’s exact test, with a false discovery rate (FDR) correction to account for multiple testing.

### RNA-Seq analysis of persister cells from exponentially growing *E. coli* DS1

To obtain enough RNA from persister cells for the RNA-Seq analysis, 6 replicate flasks, each containing 300 mL of LB media, were individually inoculated with 10 μL of an overnight culture of *E. coli* DS1 and incubated at 37°C and 200 rpm until the culture reached an OD of 0.4. After reaching the desired OD, the bacterial cultures were pelleted, immediately frozen and stored at −30°C. This was repeated until a total of 9.3 liters of exponentially grown cultures was processed for each of the two biological replicates.

To extract total RNA from exponential-phase persister cells, persisters were first isolated by employing the lysis protocol (S3 File) described in [24], and the RNA from the lysed non-persister cells was completely degraded using RNAse A (Sigma-Aldrich, St. Louis, USA) before proceeding with the total RNA extraction. The complete degradation of the RNA from the non-persister cells was determined with a gel electrophoresis of RNA extractions of the supernatant. Prior to the extraction of the total RNA from persisters, the pellets were washed three times.

Phenol-chloroform RNA extractions were performed in duplicate for both exponentially growing cells and DS1-(*hipQ*)-strain exponential-phase persisters from a pellet equivalent to an initial culture of 150 mL and 4.5 L for each biological replicate, respectively.

DNA degradation and total RNA purification were performed with a Qiagen RNeasy kit according to the manufacturer’s protocols (Qiagen, Hilden, Germany). RNA-Seq on each sample was performed at BGI Genomics on an Illumina HiSeq 2000 instrument using 2 × 101 paired-end tags and strand-specific chemistry. Raw reads were processed as indicated above.

### Transcriptome assembly, annotation, and analysis

For exponential-phase persisters and exponentially growing normal cells from the DS1 strain, we assembled the complete transcriptome using Trinity [93] for both *de novo* and genome-guided assemblies. Each transcriptome was then used for protein prediction and annotation of genes using Trinotate [93]. The Trinotate pipeline includes a homology search to known sequence data using blastx and blastp [94], the identification of protein domains using HMMER v3.0 [95], a prediction of signal peptides with SignalP [96] and tmHMM [97], and several comparisons to curated annotation databases, such as EMBL, UniProt, KEGG [98], eggNOG [99], and Gene Ontology [100].

TopHat2 was employed to map all PET reads to the reference genome [101]. Expression levels were presented as Fragments per Kilobase of exon per Million reads (FPKMs), and differential gene expression analyses were performed using both CuffDiff2 [102,103] and NOISeq [104] using the cutoffs of a p-value of 0.05 and a q of 0.9, respectively.

### Analysis of overrepresented GO terms in differentially expressed genes during persistence

To analyze the biological processes that are significantly regulated during spontaneous persistence, we performed a Blast2GO [46] analysis over the complete *E. coli* deduced proteome. We then tested for significant overrepresentation of GO terms in the groups of differentially expressed, overexpressed, and underexpressed genes derived from CuffDiff [102] and NOISeq [104] analyses performed between *E. coli* DS1 exponentially growing cells and exponential-phase persisters. The overrepresentation analysis was performed using DAVID 6.7 and Fisher’s exact test, with a false discovery rate (FDR) correction to account for multiple testing (Benjamini-Hochberg test) and a Bonferroni score threshold of < 0.005.

### RT-qPCR validation of detected differentially expressed genes

From the group of differentially expressed genes, the expression profiles of 14 genes were chosen to be validated with RT-qPCR. The selection of these genes accounted for their biological function and the existence of previous reports of their relevance to triggered persistence [2,8,28]. The housekeeping genes *dxs* and *opgH* were used to normalize the data. For this analysis, a sample of the identical RNA that was sequenced and the RNA from a biological replica for each condition were converted to cDNA using random hexamers prior to the qPCR. The cDNA synthesis and qPCR were performed with the DyNAmo SYBR Green 2-Step qRT-PCR Kit (Thermo Scientific*,* Waltham*,* USA).

The validation of the differentially expressed genes through qPCR was performed using the relative quantification method with a standard curve on a 7500 Fast qPCR Instrument (Applied Biosystems, Life Technologies, California, USA). The statistical analysis of the obtained data was done with REST [105] using the Pair Wise Fixed Reallocation Randomization Test method [105].

### Determination of total fatty acids

Lipids were extracted from *E. coli* DS1 during exponential growth and stationary phase. Exponential-phase persisters were isolated from an exponential phase culture of *E. coli* DS1 using a published protocol [24], which enriches for cells with phenotypes corresponding to spontaneous persisters in this strain.

For each of the above-mentioned samples, lipids were extracted by pelleting 60 mL of each culture (or its equivalent in cell population persister samples after the isolation protocol); the pellets were then frozen at −20°C for 2 hours. Frozen pellets were then lyophilized overnight to remove any residual water. Afterwards, samples were dispersed in a chloroform/methanol/water (3:1:1 v/v) mixture and then vortexed every 15 minutes for 4 hours. After two days, three separate phases are visually identified, where the top phase is an aqueous phase, the middle phase is a protein-rich phase, and the lower phase is the organic phase enriched in total lipids. The lower organic phase was drawn off by aspiration and collected into a clean glass tube. All glassware was cleaned using a piranha (sulfuric acid and hydrogen peroxide) protocol to remove all organic residues. Chloroform was then evaporated with a steady stream of gaseous N_2_ to form a thin film at the bottom of the test tube, and the lipids were then stored at −20°C. Extracted lipids were analyzed by gas chromatography.

Preparation of methyl esters of fatty acids (FAMEs) for analysis by gas chromatography/flame ionization detection (GC/FID) was performed as already described [106]. For acidic hydrolysis, 1 mL methanol/toluene (2:1, v/v) containing 2.75% (v/v) H_2_SO_4_ (95−97%) and 2% (v/v) dimethoxypropan was added to the dry sample material. For later quantification of the fatty acids, 20 µg triheptadecanoate (Tri-17:0) were added and the sample was incubated for 1 h at 80°C. To extract the resulting FAMEs, 200 µL of saturated aqueous NaCl solution and 2 mL of hexane were added. The hexane phase was dried under streaming nitrogen and re-dissolved with equal volumes of water and hexane. The hexane phase was filtrated with cotton wool soaked with NaSO_4_ and dried under streaming nitrogen. Finally, the sample was re-dissolved in 10 µL acetonitrile for GC analysis performed with an Agilent (Waldbronn, Germany) 6890 gas chromatograph fitted with a capillary DB-23 column (30 m x 0.25 mm; 0.25 µm coating thickness; J&W Scientific, Agilent). Helium was used as carrier gas at a flow rate of 1 mL/min. The temperature gradient was 150°C for 1 min, 150–200°C at 8 K/min, 200–250°C at 25 K/min and 250°C for 6 min. For verification of the peak identities, an aliquot of the sample was analysed by GC/mass spectrometric detection (GC/MS) using an Agilent 5973 Network mass selective detector connected to the gas chromatograph as described above. The injection temperature was 220°C. The temperature gradient as well as the carrier gas was carried out as described for the GC analysis. Electron energy of 70 eV, an ion source temperature of 230°C, and a temperature of 350°C for the transfer line were used. Mass detection was performed in scan mode in an *m*/*z* range of 50–650. Lipid extraction and analysis were perfor*m*ed for each of the above-described samples with three biological replicates.

## Supporting information

S1 File Novel SNPs detected in *E. coli* DS1. Set of 112 SNPs detected in the genome of *E. coli* DS1 against all three reference genomes.
(XLSX)

S2 File Prediction of *E. coli* DS1 HipA protein aligned to the crystal structure of HipA of MG1655. AlphaFold2 prediction made using ColabFold v1.5.2 of the HipA protein carrying the novel A242V mutation identified in the genome of *E. coli* DS1. The resultant structure (silver) was aligned to the crystal structure of HipA from MG1655 (blue) using the Pairwise Structure Alignment tool from RCSB PDB. The model for DS1’s HipA is depicted in silver, and MG1655 HipA is depicted in blue. A zoomed in view into the region of residue 242 is shown as an inset.
(PDF)

S3 File Schematic of persister isolation protocol using antibiotics or the lysis protocol. Traditional antibiotic-based protocols typically involve a short pre-growth phase (e.g., 2 hours for 100x diluted stationary cultures) in order to efficiently kill stationary phase cells, which is followed by an extended antibiotic treatment (3–5 hours). This prolonged treatment may induce stress in spontaneous persisters, potentially altering their cellular components. Additionally, it may trigger normal cells to transition into triggered persisters. In contrast, our lysis-based protocol incorporates an extended pre-growth phase followed by rapid lysis-based isolation, mitigating these confounding variables.
(PDF)

S4 File FOV of isolated persisters from a stationary culture of *E. coli* DS1. Illustrative DIC images of the sample of spontaneous persisters isolated from a stationary phase culture of the *E. coli* DS1 (*hipQ*) strain using the lysis protocol, at 7 minutes and 120 minutes post treatment. This figure was generated from images published as File S2 from Cañas-Duarte SJ et al, 2014 (PMID 24586365).
(PDF)

S5 File Transcriptome of E. coli DS1 persisters. Complete transcriptome results for exponential-phase persisters of the DS1 (*hipQ*) strain. File includes all FPKM values for all mapped genes and the classification lists of Up/Down regulated clusters.
(XLSX)

S6 File Set of differentially expressed genes for RT-qPCR validation. Set of genes found to be differentially expressed in the *hipQ* exponential-phase persisters that were chosen for validation.
(XLSX)

S7 File RT-qPCR validation results. Normalized and non-normalized calculations of the relative expression of the 12 differentially expressed genes chosen for validation.
(PDF)

S8 File SNPs directly related to the differential expression of genes in persistence. Description of data: List of genes found to contain at least one SNP that appear correlated with genes found to be differentially expressed in exponential-phase persisters in *E. coli* DS1.
(XLSX)

S9 File Complete fatty acid profile results for *E. coli* strains in different physiological conditions. Fatty acid analyses results for *E. coli* cells in exponential and stationary phase and of triggered and spontaneous persisters.
(XLSX)

S10 File Morphological characteristics of exponentially growing *E. coli* strains DS1, KL16 and MG1655 at OD600 = 0.4. Cultures were grown in 50 mL of LB at 37°C in 250 mL flasks until they reached an OD600 of 0.4. Samples were spotted on 1.5% LB-agarose pads and imaged using a 100 × Ph3 objective on a Nikon Ti2 inverted microscope at 37°C. Supersegger-Omnipose was used to segment the cells and an in-house Matlab code was used to analyze the extracted features.
(PDF)

S11 File Raw CFU counts for persister frequency quantifications. Persister frequencies were determined as described on Figure 3. Raw CFU counts are indicated for t = 0 and t = 3 hours for all reported strains.
(XLSX)
