# Supplementary material for: An integrative multi-omics approach points to membrane composition as a key factor in E. coli persistence
Source: PLoS One. 2026 Jun 29;21(6):e0351161. doi: 10.1371/journal.pone.0351161 (PMC13313352; doi:10.1371/journal.pone.0351161)
Supplement: S2 File — AlphaFold2 prediction made using ColabFold v1.5.2 of the HipA protein carrying the novel A242V mutation identified in the genome of E. coli DS1. The resultant structure (silver) was aligned to the crystal structure of HipA from MG1655 (blue) using the Pairwise Structure Alignment tool from RCSB PDB. The model for DS1’s HipA is depicted in silver, and MG1655 HipA is depicted in blue. A zoomed in view into the region of residue 242 is shown as an inset. (PDF) [file pone.0351161.s002.pdf]

Title: Prediction of *E. coli* DS1 HipA protein aligned to the crystal structure of HipA of MG1655

Legend: AlphaFold2 prediction made using ColabFold v1.5.2 [1] of the HipA protein carrying the novel A242V mutation identified in the genome of *E. coli* DS1. The resultant structure (silver) was aligned to the crystal structure of HipA from MG1655 [2] (blue) using the Pairwise Structure Alignment tool from RCSB PDB [3]. The model for DS1's HipA is depicted in silver, and MG1655 HipA is depicted in blue. A zoomed in view into the region of residue 242 is shown as an inset.

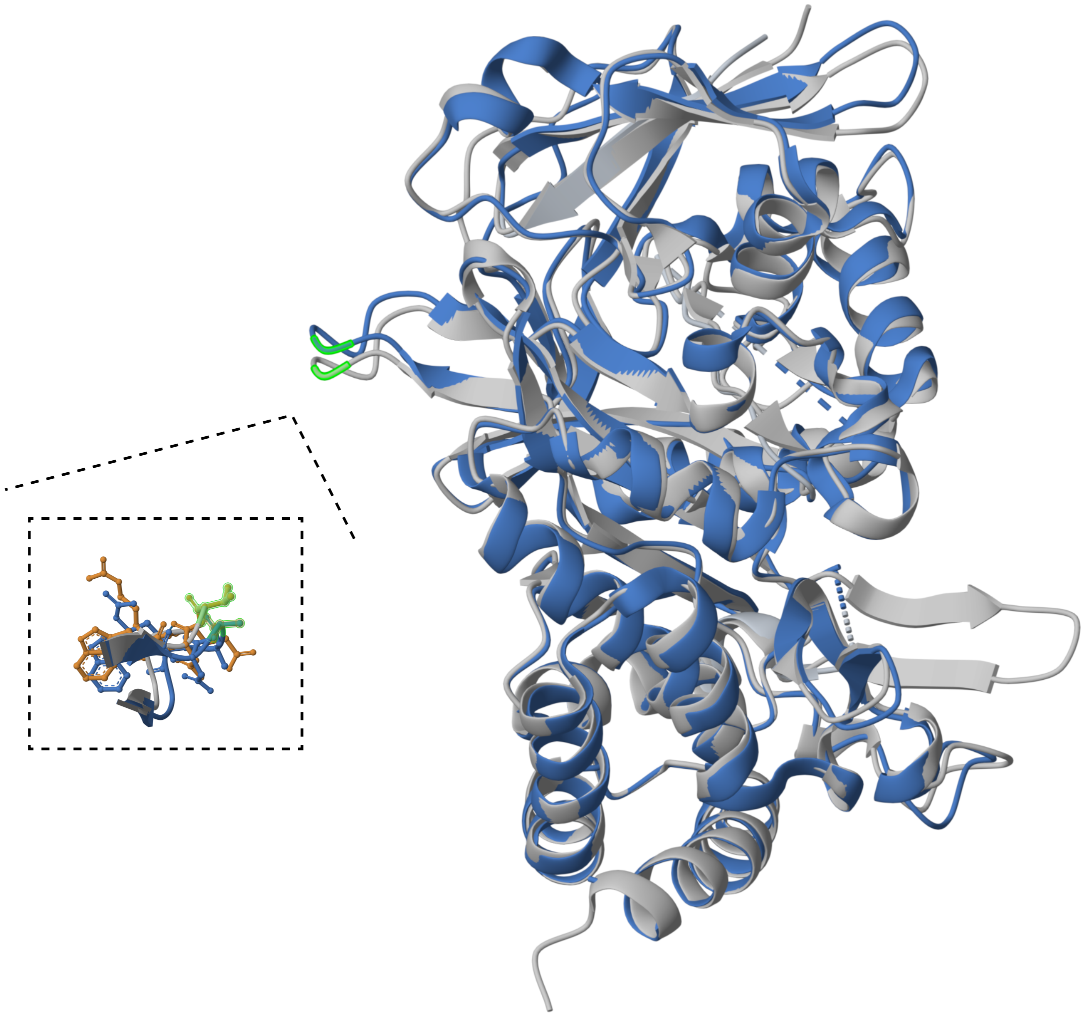

References

1. Mirdita M, Schütze K, Moriwaki Y, Heo L, Ovchinnikov S, Steinegger M. ColabFold: making protein folding accessible to all. Nat Methods. 2022;19:679–82.
2. BERNSTEIN FC, KOETZLE TF, WILLIAMS GJB, MEYER EF, BRICE MD, RODGERS JR, et al. The Protein Data Bank. A Computer-Based Archival File for Macromolecular Structures. Eur J Biochem. 1977;80:319–24.
3. Schumacher MA, Balani P, Min J, Chinnam NB, Hansen S, Vulić M, et al. HipBA–promoter structures reveal the basis of heritable multidrug tolerance. Nature. 2015;524:59–64.
