# Supplementary material for: An integrative multi-omics approach points to membrane composition as a key factor in E. coli persistence
Source: PLoS One. 2026 Jun 29;21(6):e0351161. doi: 10.1371/journal.pone.0351161 (PMC13313352; doi:10.1371/journal.pone.0351161)

Title: FOV of enriched spontaneous persisters from a stationary culture of E. coli DS1.

Legend: Illustrative DIC images of the sample of spontaneous persisters isolated from a stationary phase culture of the *E. coli* DS1 (hipQ) strain using the lysis protocol, at 7 minutes and 120 minutes post treatment. figure was generated from images published as File S2 from Cañas-Duarte SJ et al, 2014 (PMID 24586365).

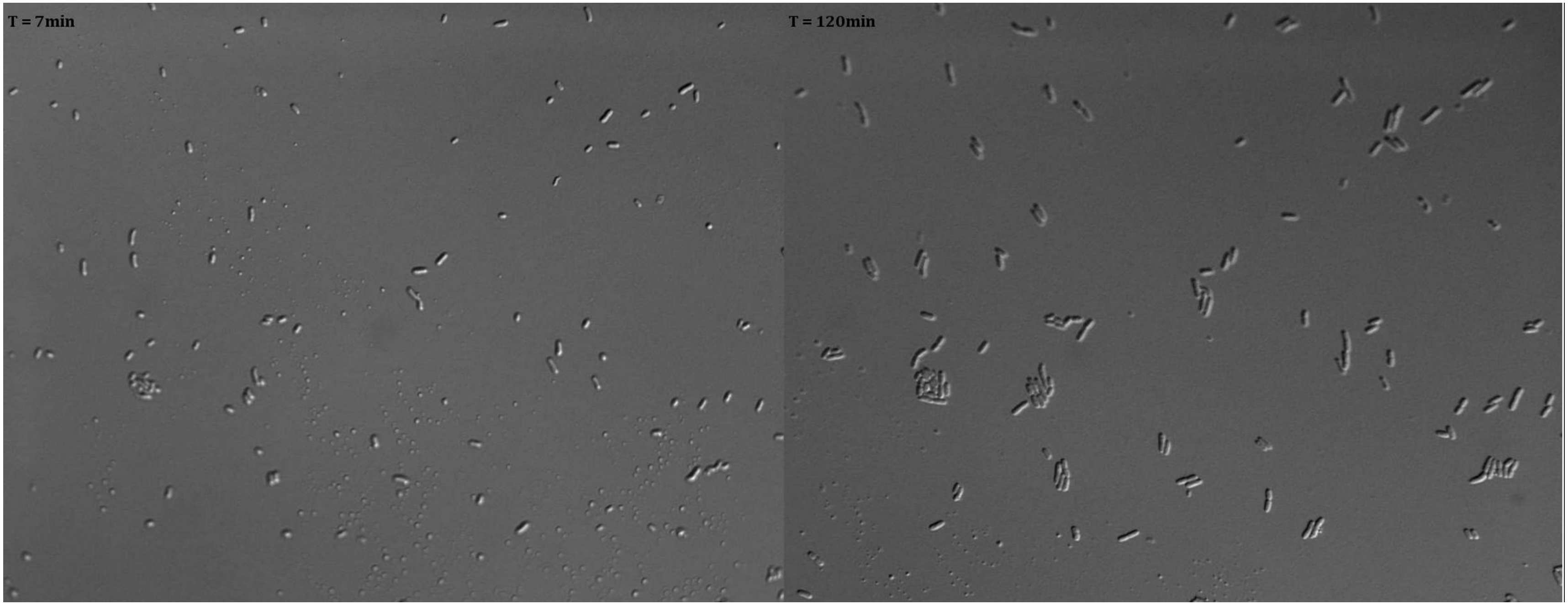

Supplement: S4 File — Illustrative DIC images of the sample of spontaneous persisters isolated from a stationary phase culture of the E. coli DS1 (hipQ) strain using the lysis protocol, at 7 minutes and 120 minutes post treatment. This figure was generated from images published as File S2 from Cañas-Duarte SJ et al, 2014 (PMID 24586365). (PDF) [file pone.0351161.s004.pdf]
