# Supplementary material for: An integrative multi-omics approach points to membrane composition as a key factor in E. coli persistence
Source: PLoS One. 2026 Jun 29;21(6):e0351161. doi: 10.1371/journal.pone.0351161 (PMC13313352; doi:10.1371/journal.pone.0351161)
Supplement: S7 File — Normalized and non-normalized calculations of the relative expression of the 12 differentially expressed genes chosen for validation. (PDF) [file pone.0351161.s007.pdf]

Title: RT-qPCR validation results
Legend: Normalized and non-normalized calculations of the relative expression of the 12 differentially expressed genes chosen for validation.

Relative Expression Results

Parameter	Value
Iterations	6000

Gene	Type	Reaction Efficiency	Expression	Std. Error	95% C.I.	P(H1)	Result
opgH	REF	1.2923	0.764				
dxs	REF	0.7966	1.309				
fadB	TRG	0.8406	5.047	2.881 - 10.588	1.873 - 12.745	0.035	UP
tisB	TRG	0.7683	0.191	0.104 - 0.512	0.059 - 0.631	0.035	DOWN
cadA	TRG	1.1603	0.058	0.033 - 0.110	0.022 - 0.140	0.035	DOWN
relB	TRG	0.9881	0.172	0.118 - 0.319	0.067 - 0.347	0.035	DOWN
dinJ	TRG	0.6368	0.401	0.270 - 0.661	0.192 - 0.760	0.019	DOWN
soxS	TRG	0.6951	4.951	3.271 - 8.067	2.935 - 9.879	0.035	UP
hde	TRG	0.6977	0.109	0.066 - 0.161	0.056 - 0.192	0.024	DOWN
acs	TRG	0.6848	2.676	1.873 - 4.125	1.455 - 4.937	0.035	UP
iraP	TRG	0.6507	0.263	0.132 - 0.539	0.081 - 0.629	0.035	DOWN
ompX	TRG	0.9019	0.125	0.067 - 0.182	0.060 - 0.243	0.035	DOWN
pspB	TRG	1.5741	22.295	11.716 - 42.509	8.006 - 50.970	0.025	UP
groS	TRG	1.5722	0.155	0.125 - 0.195	0.108 - 0.230	0.035	DOWN
yodD	TRG	1.4021	0.222	0.148 - 0.450	0.075 - 0.480	0.035	DOWN
tnaA	TRG	1.5454	8.520	3.767 - 15.132	2.890 - 32.011	0.013	UP

Interpretation

fadB is UP-regulated in sample group (in comparison to control group) by a mean factor of 5.047 (S.E. range is 2.881 - 1
fadB sample group is different to control group. P(H1)=0.035

tisB is DOWN-regulated in sample group (in comparison to control group) by a mean factor of 0.191 (S.E. range is 0.104
tisB sample group is different to control group. P(H1)=0.035

cadA is DOWN-regulated in sample group (in comparison to control group) by a mean factor of 0.058 (S.E. range is 0.03
cadA sample group is different to control group. P(H1)=0.035

relB is DOWN-regulated in sample group (in comparison to control group) by a mean factor of 0.172 (S.E. range is 0.118
relB sample group is different to control group. P(H1)=0.035

dinJ is DOWN-regulated in sample group (in comparison to control group) by a mean factor of 0.401 (S.E. range is 0.270
dinJ sample group is different to control group. P(H1)=0.019

soxS is UP-regulated in sample group (in comparison to control group) by a mean factor of 4.951 (S.E. range is 3.271 - [illegible]
soxS sample group is different to control group. P(H1)=0.035

hde is DOWN-regulated in sample group (in comparison to control group) by a mean factor of 0.109 (S.E. range is 0.066
hde sample group is different to control group. P(H1)=0.024

acs is UP-regulated in sample group (in comparison to control group) by a mean factor of 2.676 (S.E. range is 1.873 - 4.[illegible]
acs sample group is different to control group. P(H1)=0.035

iraP is DOWN-regulated in sample group (in comparison to control group) by a mean factor of 0.263 (S.E. range is 0.132
iraP sample group is different to control group. P(H1)=0.035

ompX is DOWN-regulated in sample group (in comparison to control group) by a mean factor of 0.125 (S.E. range is 0.0[illegible]
ompX sample group is different to control group. P(H1)=0.035

pspB is UP-regulated in sample group (in comparison to control group) by a mean factor of 22.295 (S.E. range is 11.716
pspB sample group is different to control group. P(H1)=0.025

groS is DOWN-regulated in sample group (in comparison to control group) by a mean factor of 0.155 (S.E. range is 0.12[illegible]
groS sample group is different to control group. P(H1)=0.035

yodD is DOWN-regulated in sample group (in comparison to control group) by a mean factor of 0.222 (S.E. range is 0.14
yodD sample group is different to control group. P(H1)=0.035

tnaA is UP-regulated in sample group (in comparison to control group) by a mean factor of 8.520 (S.E. range is 3.767 - 1

tnaA sample group is different to control group. P(H1)=0.013

Non-Normalised Results

Gene	Type	Reaction Efficiency	Expression	Std. Error	95% C.I.	P(H1)	Result
opgH	REF	1.2923	0.813	0.677 - 0.978	0.607 - 1.112	0.263	
dxs	REF	0.7966	1.392	0.636 - 3.618	0.462 - 5.107	0.465	
fadB	TRG	0.8406	5.369	4.631 - 6.579	4.343 - 7.045	0.000	UP
tisB	TRG	0.7683	0.203	0.118 - 0.336	0.116 - 0.346	0.048	DOWN
cadA	TRG	1.1603	0.062	0.053 - 0.071	0.051 - 0.081	0.000	DOWN
relB	TRG	0.9881	0.183	0.144 - 0.231	0.137 - 0.245	0.000	DOWN
dinJ	TRG	0.6368	0.427	0.382 - 0.462	0.370 - 0.472	0.000	DOWN
soxS	TRG	0.6951	5.267	4.210 - 6.829	3.881 - 7.648	0.048	UP
hde	TRG	0.6977	0.116	0.102 - 0.143	0.099 - 0.147	0.000	DOWN
acs	TRG	0.6848	2.847	2.337 - 3.452	2.282 - 3.504	0.035	UP
iraP	TRG	0.6507	0.279	0.214 - 0.362	0.182 - 0.373	0.030	DOWN
ompX	TRG	0.9019	0.133	0.107 - 0.152	0.103 - 0.185	0.010	DOWN
pspB	TRG	1.5741	23.717	15.682 - 30.956	14.412 - 49.322	0.000	UP
groS	TRG	1.5722	0.164	0.100 - 0.277	0.076 - 0.378	0.000	DOWN
yodD	TRG	1.4021	0.236	0.182 - 0.348	0.149 - 0.388	0.000	DOWN
tnaA	TRG	1.5454	9.063	5.878 - 18.211	3.065 - 20.524	0.000	UP
