# Supplementary material for: An integrative multi-omics approach points to membrane composition as a key factor in E. coli persistence
Source: PLoS One. 2026 Jun 29;21(6):e0351161. doi: 10.1371/journal.pone.0351161 (PMC13313352; doi:10.1371/journal.pone.0351161)
Supplement: S10 File — Cultures were grown in 50 mL of LB at 37°C in 250 mL flasks until they reached an OD600 of 0.4. Samples were spotted on 1.5% LB-agarose pads and imaged using a 100 × Ph3 objective on a Nikon Ti2 inverted microscope at 37°C. Supersegger-Omnipose was used to segment the cells and an in-house Matlab code was used to analyze the extracted features. (PDF) [file pone.0351161.s010.pdf]

Title: Morphological characteristics of exponentially growing *E. coli* strains DS1, KL16 and MG1655 at OD600 =0.4

Legend: Cultures were grown in 50 mL of LB at 37C in 250 mL flasks until they reached an OD600 of 0.4. Samples were spotted in 1.5% LB-agarose pads and imaged using a 100x Ph3 objective in a Nikon Ti2 inverted microscope at 37C. Supersegger-Omnipose [1-3] was used to segment the cells and an inhouse Matlab code was used to analyze the extracted features.

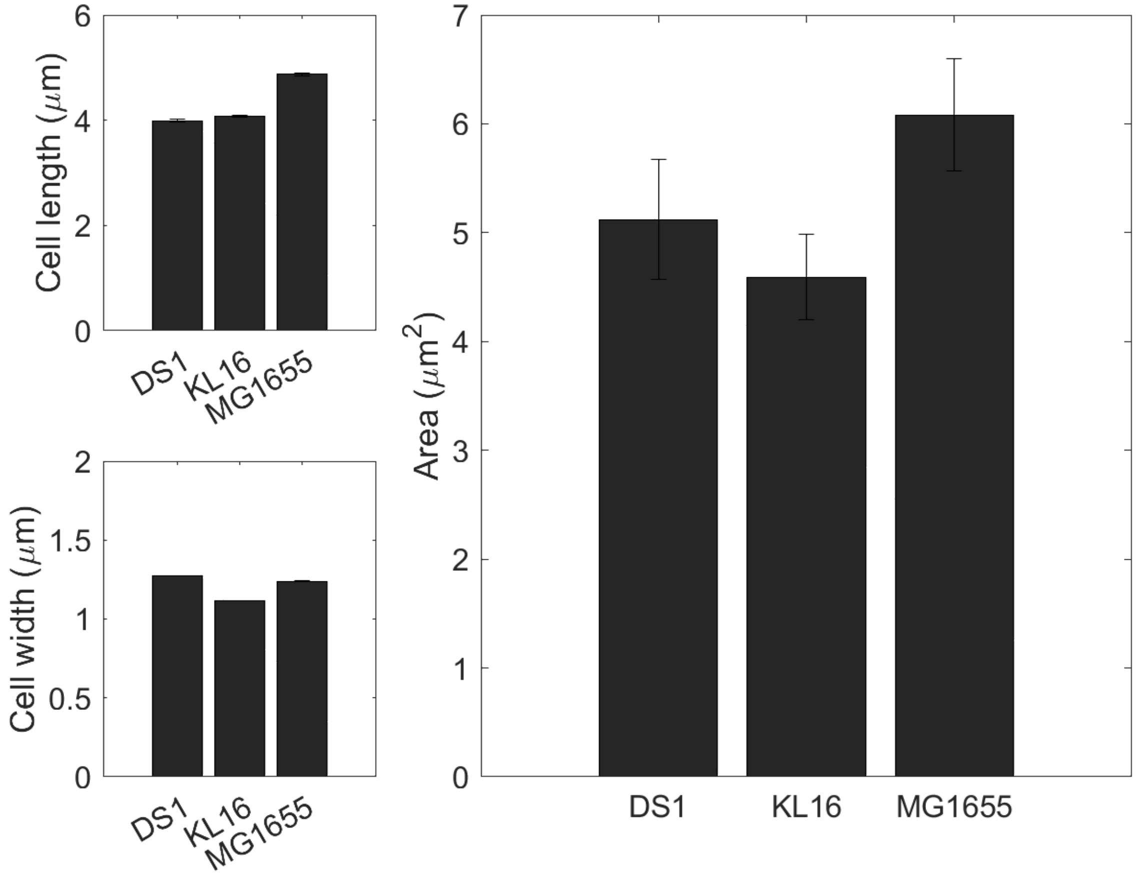

References

1. Cass JA, Stylianidou S, Kuwada NJ, Traxler B, Wiggins PA. Probing bacterial cell biology using image cytometry. Mol Microbiol. 2017;103:818–28.
2. Cutler KJ, Stringer C, Lo TW, Rappez L, Stroustrup N, Brook Peterson S, et al. Omnipose: a high-precision morphology-independent solution for bacterial cell segmentation. Nat Methods. 2022;19:1438–48.
3. Stylianidou S, Brennan C, Nissen SB, Kuwada NJ, Wiggins PA. SuperSegger: robust image segmentation, analysis and lineage tracking of bacterial cells. Mol Microbiol. 2016;102:690–700.
