# Supplementary material for: An integrative multi-omics approach points to membrane composition as a key factor in E. coli persistence
Source: PLoS One. 2026 Jun 29;21(6):e0351161. doi: 10.1371/journal.pone.0351161 (PMC13313352; doi:10.1371/journal.pone.0351161)
Supplement: Attachment — Submitted filename: Rebuttal_PloSOne_20260115.docx [file pone.0351161.s012.docx]

Reviewer #1: In this manuscript, the authors use a multi-omics approach to study so-called triggered and spontaneous persisters in E. coli, focusing on genome sequencing of the DS1 mutant, transcriptomic comparisons between exponential-phase survivors of their chemical lysis protocol and non-persisters, and lipidomic profiling across different strains and growth conditions. They conclude that membrane remodeling, rather than canonical stress responses, underlies persistence and contributes to multitolerance. I have some major comments as follows:

We thank the reviewer for its comments and their thoughtful consideration of our study.

1. The manuscript needs substantial rewording and clarification regarding the use of the terms “spontaneous” and “triggered” persisters. Although the hipQ (DS1) and hipA7 mutants are well-known high-persistence strains, it is not clear that they can be definitively classified into these two categories. In the exponential-phase hipQ cultures analyzed here, the surviving population is likely to be highly heterogeneous... some persisters may indeed form stochastically as slow growers, but others may represent carryover from overnight cultures. These possibilities are not examined, and spontaneous persister formation is not clearly verified in the present study. The frequently cited study by Balaban et al. relied on microscopy, which by its nature tracks only a limited number of cells and therefore cannot capture the full complexity of persister heterogeneity. Rather than framing the work around a strict division between “spontaneous” and “triggered” persisters, the authors should align their terminology more closely with their experimental approach, for example by describing “persisters obtained from DS1 in exponential phase” or “persisters arising from stationary-phase hipA7.” I also recommend removing Figure 1, which presents an overly simplified view of a complex and heterogeneous phenomenon. Persisters can arise through multiple routes, including carryover from stationary phase, formation during overnight culture, stochastic events in exponential growth, or responses to stresses, and this complexity should be acknowledged rather than reduced to two categories.

We appreciate the suggestion for more carefully wording our findings and have edited the text accordingly. Regarding the classification of the two strains, we are using that of the main type of persistence for which they are known for. As shown in our previous work, we acknowledge that persisters of both types can (and do) coexist under different conditions in not only the wild type E. coli strain but also on the DS1 and HipA7 strains. To diminish the influence of this complexity, we made sure to use a high dilution from (>1:30000) in all our exponential experiments to minimize the carryover of triggered persisters from the preceding stationary phase culture. As shown in Fig 6 and the supplementary photos from the protocol paper (PMID: 24586365), the isolated spontaneous persisters from the hipQ (DS1) strain exhibit the continuous and slow growth characteristic of their type, and < 5% of the spotted aliquot after persister isolation are yet to resume growth after 2 hours indicating a potentially low contamination with potential VBNCs even in stationary phase.

Overall, while we agree that the isolated persister population is likely heterogeneous, in the context of DS1/*hipQ* with approximately 100-fold higher spontaneous persister frequency, available data suggests the isolates are overwhelmingly dominated by spontaneous persisters, just as intended.

2. It is surprising that the genomic analysis is restricted to SNPs. Whole genome sequencing, particularly with the Illumina HiSeq platform and the analysis tools described, is also capable of identifying small insertions/deletions and in some cases larger chromosomal rearrangements or structural abnormalities. The authors do not explain why these classes of variation were not analyzed or reported. They should clarify whether such variants were detected and, if so, provide a rationale for why they were excluded from the study. If they did not identify them, then they should explain the limitations of their pipeline.

We apologize for the confusion. The analysis we performed included detection of small indels (insertions/deletions), as well as the whole assembly and annotation of the genome of the DS1 strain. We have modified the text to make it clearer. Whole genome alignment performed using progressiveMauve between the DS1 and KLY genomes also failed to reveal any indications of genome rearrangements or large-scale structural changes.

3. The authors compare exponential-phase DS1 cultures without treatment to the subpopulation of cells that survived their previously described chemical lysis protocol. I find this approach problematic. The chemical treatment does not necessarily select for bona fide persisters; rather, it may enrich for cells with altered membranes that resist lysis, or it may artificially induce dormancy and thereby confer antibiotic tolerance. The RNA-seq results presented by the authors in fact suggest that this possibility cannot be excluded. A stronger and more convincing approach would have been to directly treat exponential-phase cultures with ampicillin and perform transcriptomic analysis on the surviving unlysed cells. In doing so, the authors should avoid prolonged overnight cultures, as these can increase the frequency of VBNC cells. If exponential-phase cultures are free of VBNC, then antibiotic treatment should mainly enrich for persisters. This can be validated by quantifying survivors with flow cytometry and comparing those results with CFU measurements; matching values would confirm the persister enrichment, whereas discrepancies would indicate VBNC contamination. In addition, the possibility that VBNC cells are being isolated by the lysis method should be explicitly addressed, and the authors should provide verification that VBNC populations are not confounding their transcriptomic analyses.

We understand the concerns raised by the reviewer regarding the nature of the isolated cells and their identity as persisters. As previously published, the protocol used isolates cells that phenotypically exhibit the characteristics of each persister type (as shown by microscopy) in addition to corresponding to a similar fraction of the population as of that of the antibiotic isolated persisters published by other studies.

For example, in the previous published methods paper, when isolating spontaneous persisters in DS1/*hipQ* strain, >95% of the isolated cells exhibited slow growth with division rates characteristic of the DS1 spontaneous persisters. As a comparison, we also isolated persisters in the *hipA7* strain, which is known to contain an elevated fraction of triggered persisters with extended dormancy before resuming regular, fast growth. In the *hipA7* isolates, none of the enriched cells displayed the uniformly slow growth as was shown after isolating persisters in DS1/*hipQ*. But we did observe isolated *hipA7* cells that suddenly resumed fast growth after extended dormancy (as expected for triggered persisters), which were not present for DS1/*hipQ* post-enrichment. To summarize these observations, available evidence suggests that the protocol most likely works as intended, it is unlikely that the protocol isolated something else that also happen to have the identical characteristics for both persister types.

Since the choice of our protocol raised so many questions, we decided to include some microscopic images in the supplementary section for explanatory purposes.

Although we would agree with the proposed post-treatment with ampicillin after isolation, the length of the antibiotic treatment allows for persisters to stocastically exit the persister state, significantly reducing the already limiting amount of persisters that could be analyzed. Further, as antibiotics are known to induce stress response, especially with such a lengthy treatment period, we worried it might generate further confounding factors.

In terms of preventing the accumulation of VBNCs, as noted before, we not only used a high dilution ratio for our exponential cultures, but also only used short stationary (<16 hours) cultures as started cultures. Although it is highly possible that a small fraction of VBNCs are present in the enriched samples of spontaneous persisters, as shown in the microscopy images of the protocol article (Fig 6 and SI) this fraction is likely negligible (< 5%).

4. The study does not go beyond descriptive transcriptomics. The authors did not perform any genetic manipulations (such as deletion of downregulated genes or overexpression of upregulated genes) to test whether these changes causally influence persistence. Such validation would be essential to move from correlation to mechanism.

This is correct, this study’s scope has aimed to help elucidate potential contributing mechanisms to spontaneous persisters as well as to help narrow down which mutations could be responsible for the hipQ phenotype of DS1. Further testing on a mechanistic cause is the subject of our next study.

5. The lipidomics study design is problematic because the authors use different strains and growth conditions, which makes direct comparisons difficult to interpret. According to their methods, lipids were extracted from E. coli MG1655 and DS1 during both exponential and stationary phase, while “triggered” and “spontaneous” persister cells were isolated from a stationary-phase culture of E. coli TH1269 and an exponential-phase culture of E. coli DS1, respectively. These conditions are not equivalent and introduce multiple variables, such as strain background, growth phase, and isolation protocol, that complicate the attribution of lipidomic differences specifically to persister physiology. I would have expected the authors to use consistent conditions and treatments across all three strains to allow for clearer and more meaningful comparisons.

We thank the reviewer for this comment and apologize for the confusion. We have now removed the lipidomic results relating to the triggered persisters, which we understand now were generating confusion. However, we would like to explain our rationale for the experimental design used. Our design was focused on separately observing the changes in each persister type compared to the strain from which they were generated both in exponential and in stationary phase. For example, for DS1 spontaneous persisters that are generated in exponential phase, we compared their lipid profiles to those of DS1 exponentially growing cells and DS1 stationary phase cells. Similarly, for the triggered persisters, we isolated persisters from MG1655 hipA7 (TH1269) in stationary phase and compared those profiles to those of MG1655 in exponential and stationary phase. As TH1629 only differs from MG1655 in a point mutation on the HipA protein, we used the MG1655 background more cleanly to observe the stationary phase profiles, given the high frequency of triggered persisters present on these conditions.

Our intent was to observe the normal range of change in the fatty acid profiles between exponential and stationary phase for each strain separately and noted how the change was more drastic when comparing each persister state to their respective “background”. However, as this seems to generate confusion, we have removed the lipidomic analysis of triggered persisters from the manuscript.
