# Supplementary material for: An integrative multi-omics approach points to membrane composition as a key factor in E. coli persistence
Source: PLoS One. 2026 Jun 29;21(6):e0351161. doi: 10.1371/journal.pone.0351161 (PMC13313352; doi:10.1371/journal.pone.0351161)
Supplement: Attachment — Submitted filename: ResponseToReviewers_20260425.docx [file pone.0351161.s013.docx]

Reviewer #1: While I appreciate the authors’ detailed responses, several concerns remain at the level of framing and language rather than experimental design (I am not requesting additional experiments at this point).

However, I strongly recommend further editing of the manuscript text to better align conclusions with what is directly supported by the data:

1) The manuscript continues to rely on a strict “spontaneous vs triggered” classification and frequently refers to the isolated DS1 population as spontaneous persisters. Given the heterogeneity acknowledged by the authors and the nature of the isolation protocol, the text should consistently use more descriptive language such as “persisters isolated from DS1 during exponential phase” rather than categorical labels, including in the Abstract and Discussion.

We thank the reviewer for their comments and thoughtful consideration of our study. We appreciate the suggestion for more careful wording throughout the text, and we have used the recommended description where appropriate.

We understand that the concern here is that although DS1 is widely acknowledged as a model organism for spontaneous persistence, the isolated population after the enrichment protocol is not guaranteed to be 100% pure. To address this, the use of “spontaneous persisters” is now largely limited to conceptual context. We have also included an explicit description of the precautions taken to minimize the fraction of non-spontaneous persisters in the isolated samples analyzed.

2) Statements implying that spontaneous persisters are uniformly slow-growing should be corrected. Spontaneous persisters do not have to be slow-growing; they can also be non-growing, and cells can stochastically enter non-growing states during exponential growth. The text as well as Figure 1 should reflect this broader and more established view of persister physiology.

We thank the reviewer for this insightful suggestion. We agree that the growth dynamics (or lack thereof) of spontaneous persisters may not be universally slow-growing outside of the *E. coli* strains for which this growth phenotype has been described. As such, we have revised the text and the Figure 1 legend accordingly to constrain this description to the DS1 strain.

We would like, however, to provide some context for our original intention. The characterization of spontaneous (Type II) persisters as slow-growing is based on the seminal work by Balaban and coauthors in 2004 with the DS1 (hipQ) strain, the canonical model in which this persister type was discovered and observed to continue growing and dividing slowly during antibiotic treatment (PMIDs: 15308767, 24586365). We recognize, however, that the growth behavior of spontaneous persisters in other strains or species has simply not been investigated in detail. This slow-growth phenotype may be specific to the few *E. coli* strains for which this type of data has been reported. The 2019 joint review by Balaban et al. (PMID: 30980069), which represents the current consensus definition of persister types, notably does not specify any particular growth trait for spontaneous persisters. Interestingly, we recently had a chat with Prof. Dan Andersson, a co-author of that review. He was surprised to hear that spontaneous persisters in DS1/hipQ actually exhibit growth. This is a strong sign that this nuance is not widely appreciated in the field.

In the revised manuscript, we have therefore kept the description that spontaneous persisters in DS1 are slow-growing, but also carefully rephrased the general description to be open to the possibility that spontaneous persisters in other strains or species could display different growth characteristics, including non-growth. We hope this now accurately reflects both the original observations and the broader perspective raised by the reviewer.

3) The manuscript repeatedly implies a causal or mechanistic role for membrane composition in persister formation, although the data support only association. The text should be revised throughout to remove causal/mechanistic implications and instead describe membrane changes as correlated with or associated with persistence.

We thank the reviewer for this comment and have modified the text accordingly. Specifically, we have changed the language referring to the potential role of the membrane in persistence to indicate correlation and involvement rather than causality.

4) The Discussion should explicitly acknowledge that the chemical lysis-based enrichment could preferentially select for cells with altered membrane properties or other non-persister cells.

We thank the reviewer for this suggestion and have now added this acknowledgement in the discussion.
