# Supplementary material for: An integrative multi-omics approach points to membrane composition as a key factor in E. coli persistence
Source: PLoS One. 2026 Jun 29;21(6):e0351161. doi: 10.1371/journal.pone.0351161 (PMC13313352; doi:10.1371/journal.pone.0351161)
Supplement: Peer review history

# Peer review: Decision Letter 0

**DOI:** 10.1371/journal.pone.0351161.r001

**Authors:** Bashir Sajo Mienda

17 Oct 2025

PONE-D-25-43801

An integrative multi-omics approach points to membrane composition as a key factor in E. coli persistence

PLOS ONE

Dear Dr. Canas-Duarte,

Thank you for submitting your manuscript to PLOS ONE. After careful consideration, we feel that it has merit but does not fully meet PLOS ONE’s publication criteria as it currently stands. Therefore, we invite you to submit a revised version of the manuscript that addresses the points raised during the review process.

Please submit your revised manuscript by Dec 01 2025 11:59PM**. I** f you will need more time than this to complete your revisions, please reply to this message or contact the journal office at plosone@plos.org. When you're ready to submit your revision, log on to https://www.editorialmanager.com/pone/ and select the 'Submissions Needing Revision' folder to locate your manuscript file.

Please include the following items when submitting your revised manuscript:

- A rebuttal letter that responds to each point raised by the academic editor and reviewer(s). You should upload this letter as a separate file labeled 'Response to Reviewers'.
- A marked-up copy of your manuscript that highlights changes made to the original version. You should upload this as a separate file labeled 'Revised Manuscript with Track Changes'.
- An unmarked version of your revised paper without tracked changes. You should upload this as a separate file labeled 'Manuscript'.

If you would like to make changes to your financial disclosure, please include your updated statement in your cover letter. Guidelines for resubmitting your figure files are available below the reviewer comments at the end of this letter.

If applicable, we recommend that you deposit your laboratory protocols in protocols.io to enhance the reproducibility of your results. Protocols.io assigns your protocol its own identifier (DOI) so that it can be cited independently in the future. For instructions see: https://journals.plos.org/plosone/s/submission-guidelines#loc-laboratory-protocols. Additionally, PLOS ONE offers an option for publishing peer-reviewed Lab Protocol articles, which describe protocols hosted on protocols.io. Read more information on sharing protocols at https://plos.org/protocols?utm_medium=editorial-email&utm_source=authorletters&utm_campaign=protocols.

We look forward to receiving your revised manuscript.

Kind regards,

Bashir Sajo Mienda, PhD

Academic Editor

PLOS ONE

Journal requirements:

When submitting your revision, we need you to address these additional requirements.

1. Please ensure that your manuscript meets PLOS ONE's style requirements, including those for file naming. The PLOS ONE style templates can be found at

https://journals.plos.org/plosone/s/file?id=wjVg/PLOSOne_formatting_sample_main_body.pdf and

https://journals.plos.org/plosone/s/file?id=ba62/PLOSOne_formatting_sample_title_authors_affiliations.pdf

2. Thank you for stating the following financial disclosure:

“Colciencias grant 120465843511 and Convocatoria de Programas de Investigación 2012 of Universidad de los Andes (SJCD, JMP, SR)

Early Postdoc Mobility fellowship from the Swiss National Science Foundation with grant number P2EZP3_184252 and P400PB_199262 (LS).”

Please state what role the funders took in the study.  If the funders had no role, please state: "The funders had no role in study design, data collection and analysis, decision to publish, or preparation of the manuscript."

If this statement is not correct you must amend it as needed.

Please include this amended Role of Funder statement in your cover letter; we will change the online submission form on your behalf.

3. Thank you for stating the following in the Funding Section of your manuscript:

“We acknowledge funding from Colciencias through grant 120465843511 and Convocatoria de Programas de Investigación 2012 of Universidad de los Andes. One of the co-authors, Lei Sun, is funded by an Early Postdoc Mobility fellowship from the Swiss National Science Foundation with grant number P2EZP3_184252 and P400PB_199262.”

We note that you have provided funding information that is currently declared in your Funding Statement. However, funding information should not appear in the Acknowledgments section or other areas of your manuscript. We will only publish funding information present in the Funding Statement section of the online submission form.

Please remove any funding-related text from the manuscript and let us know how you would like to update your Funding Statement. Currently, your Funding Statement reads as follows:

“Colciencias grant 120465843511 and Convocatoria de Programas de Investigación 2012 of Universidad de los Andes (SJCD, JMP, SR)

Please include your amended statements within your cover letter; we will change the online submission form on your behalf.

4. Please note that your Data Availability Statement is currently missing a direct link to access each database. If your manuscript is accepted for publication, you will be asked to provide these details on a very short timeline. We therefore suggest that you provide this information now, though we will not hold up the peer review process if you are unable.

5. We note that you have included the phrase “data not shown” in your manuscript. Unfortunately, this does not meet our data sharing requirements. PLOS does not permit references to inaccessible data. We require that authors provide all relevant data within the paper, Supporting Information files, or in an acceptable, public repository. Please add a citation to support this phrase or upload the data that corresponds with these findings to a stable repository (such as Figshare or Dryad) and provide and URLs, DOIs, or accession numbers that may be used to access these data. Or, if the data are not a core part of the research being presented in your study, we ask that you remove the phrase that refers to these data.

If the reviewer comments include a recommendation to cite specific previously published works, please review and evaluate these publications to determine whether they are relevant and should be cited. There is no requirement to cite these works unless the editor has indicated otherwise.

[Note: HTML markup is below. Please do not edit.]

Reviewers' comments:

Reviewer's Responses to Questions

**Comments to the Author**

1. Is the manuscript technically sound, and do the data support the conclusions?

The manuscript must describe a technically sound piece of scientific research with data that supports the conclusions. Experiments must have been conducted rigorously, with appropriate controls, replication, and sample sizes. The conclusions must be drawn appropriately based on the data presented.

Reviewer #1: Partly

**********

2. Has the statistical analysis been performed appropriately and rigorously?

Reviewer #1: Yes

**********

3. Have the authors made all data underlying the findings in their manuscript fully available?

The PLOS Data policy requires authors to make all data underlying the findings described in their manuscript fully available without restriction, with rare exception (please refer to the Data Availability Statement in the manuscript PDF file). The data should be provided as part of the manuscript or its supporting information, or deposited to a public repository. For example, in addition to summary statistics, the data points behind means, medians and variance measures should be available. If there are restrictions on publicly sharing data—e.g. participant privacy or use of data from a third party—those must be specified.

Reviewer #1: Yes

**********

4. Is the manuscript presented in an intelligible fashion and written in standard English?

PLOS ONE does not copyedit accepted manuscripts, so the language in submitted articles must be clear, correct, and unambiguous. Any typographical or grammatical errors should be corrected at revision, so please note any specific errors here.

Reviewer #1: Yes

**********

5. Review Comments to the Author

Please use the space provided to explain your answers to the questions above. You may also include additional comments for the author, including concerns about dual publication, research ethics, or publication ethics. (Please upload your review as an attachment if it exceeds 20,000 characters)

Reviewer #1: In this manuscript, the authors use a multi-omics approach to study so-called triggered and spontaneous persisters in E. coli, focusing on genome sequencing of the DS1 mutant, transcriptomic comparisons between exponential-phase survivors of their chemical lysis protocol and non-persisters, and lipidomic profiling across different strains and growth conditions. They conclude that membrane remodeling, rather than canonical stress responses, underlies persistence and contributes to multitolerance. I have some major comments as follows:

1. The manuscript needs substantial rewording and clarification regarding the use of the terms “spontaneous” and “triggered” persisters. Although the hipQ (DS1) and hipA7 mutants are well-known high-persistence strains, it is not clear that they can be definitively classified into these two categories. In the exponential-phase hipQ cultures analyzed here, the surviving population is likely to be highly heterogeneous... some persisters may indeed form stochastically as slow growers, but others may represent carryover from overnight cultures. These possibilities are not examined, and spontaneous persister formation is not clearly verified in the present study. The frequently cited study by Balaban et al. relied on microscopy, which by its nature tracks only a limited number of cells and therefore cannot capture the full complexity of persister heterogeneity. Rather than framing the work around a strict division between “spontaneous” and “triggered” persisters, the authors should align their terminology more closely with their experimental approach, for example by describing “persisters obtained from DS1 in exponential phase” or “persisters arising from stationary-phase hipA7.” I also recommend removing Figure 1, which presents an overly simplified view of a complex and heterogeneous phenomenon. Persisters can arise through multiple routes, including carryover from stationary phase, formation during overnight culture, stochastic events in exponential growth, or responses to stresses, and this complexity should be acknowledged rather than reduced to two categories.

2. It is surprising that the genomic analysis is restricted to SNPs. Whole genome sequencing, particularly with the Illumina HiSeq platform and the analysis tools described, is also capable of identifying small insertions/deletions and in some cases larger chromosomal rearrangements or structural abnormalities. The authors do not explain why these classes of variation were not analyzed or reported. They should clarify whether such variants were detected and, if so, provide a rationale for why they were excluded from the study. If they did not identify them, then they should explain the limitations of their pipeline.

3. The authors compare exponential-phase DS1 cultures without treatment to the subpopulation of cells that survived their previously described chemical lysis protocol. I find this approach problematic. The chemical treatment does not necessarily select for bona fide persisters; rather, it may enrich for cells with altered membranes that resist lysis, or it may artificially induce dormancy and thereby confer antibiotic tolerance. The RNA-seq results presented by the authors in fact suggest that this possibility cannot be excluded. A stronger and more convincing approach would have been to directly treat exponential-phase cultures with ampicillin and perform transcriptomic analysis on the surviving unlysed cells. In doing so, the authors should avoid prolonged overnight cultures, as these can increase the frequency of VBNC cells. If exponential-phase cultures are free of VBNC, then antibiotic treatment should mainly enrich for persisters. This can be validated by quantifying survivors with flow cytometry and comparing those results with CFU measurements; matching values would confirm the persister enrichment, whereas discrepancies would indicate VBNC contamination. In addition, the possibility that VBNC cells are being isolated by the lysis method should be explicitly addressed, and the authors should provide verification that VBNC populations are not confounding their transcriptomic analyses.

4. The study does not go beyond descriptive transcriptomics. The authors did not perform any genetic manipulations (such as deletion of downregulated genes or overexpression of upregulated genes) to test whether these changes causally influence persistence. Such validation would be essential to move from correlation to mechanism.

5. The lipidomics study design is problematic because the authors use different strains and growth conditions, which makes direct comparisons difficult to interpret. According to their methods, lipids were extracted from E. coli MG1655 and DS1 during both exponential and stationary phase, while “triggered” and “spontaneous” persister cells were isolated from a stationary-phase culture of E. coli TH1269 and an exponential-phase culture of E. coli DS1, respectively. These conditions are not equivalent and introduce multiple variables, such as strain background, growth phase, and isolation protocol, that complicate the attribution of lipidomic differences specifically to persister physiology. I would have expected the authors to use consistent conditions and treatments across all three strains to allow for clearer and more meaningful comparisons.

**********

6. PLOS authors have the option to publish the peer review history of their article (what does this mean?). If published, this will include your full peer review and any attached files.

If you choose “no”, your identity will remain anonymous but your review may still be made public.

**Do you want your identity to be public for this peer review?**  For information about this choice, including consent withdrawal, please see our Privacy Policy.

Reviewer #1: No

**********

[NOTE: If reviewer comments were submitted as an attachment file, they will be attached to this email and accessible via the submission site. Please log into your account, locate the manuscript record, and check for the action link "View Attachments". If this link does not appear, there are no attachment files.]

While revising your submission, please upload your figure files to the Preflight Analysis and Conversion Engine (PACE) digital diagnostic tool, https://pacev2.apexcovantage.com/. PACE helps ensure that figures meet PLOS requirements. To use PACE, you must first register as a user. Registration is free. Then, login and navigate to the UPLOAD tab, where you will find detailed instructions on how to use the tool. If you encounter any issues or have any questions when using PACE, please email PLOS at figures@plos.org. Please note that Supporting Information files do not need this step.

# Author comment: Author response to Decision Letter 1

**DOI:** 10.1371/journal.pone.0351161.r002

15 Jan 2026

Reviewer #1: In this manuscript, the authors use a multi-omics approach to study so-called triggered and spontaneous persisters in E. coli, focusing on genome sequencing of the DS1 mutant, transcriptomic comparisons between exponential-phase survivors of their chemical lysis protocol and non-persisters, and lipidomic profiling across different strains and growth conditions. They conclude that membrane remodeling, rather than canonical stress responses, underlies persistence and contributes to multitolerance. I have some major comments as follows:

We thank the reviewer for its comments and their thoughtful consideration of our study.

1. The manuscript needs substantial rewording and clarification regarding the use of the terms “spontaneous” and “triggered” persisters. Although the hipQ (DS1) and hipA7 mutants are well-known high-persistence strains, it is not clear that they can be definitively classified into these two categories. In the exponential-phase hipQ cultures analyzed here, the surviving population is likely to be highly heterogeneous... some persisters may indeed form stochastically as slow growers, but others may represent carryover from overnight cultures. These possibilities are not examined, and spontaneous persister formation is not clearly verified in the present study. The frequently cited study by Balaban et al. relied on microscopy, which by its nature tracks only a limited number of cells and therefore cannot capture the full complexity of persister heterogeneity. Rather than framing the work around a strict division between “spontaneous” and “triggered” persisters, the authors should align their terminology more closely with their experimental approach, for example by describing “persisters obtained from DS1 in exponential phase” or “persisters arising from stationary-phase hipA7.” I also recommend removing Figure 1, which presents an overly simplified view of a complex and heterogeneous phenomenon. Persisters can arise through multiple routes, including carryover from stationary phase, formation during overnight culture, stochastic events in exponential growth, or responses to stresses, and this complexity should be acknowledged rather than reduced to two categories.

We appreciate the suggestion for more carefully wording our findings and have edited the text accordingly. Regarding the classification of the two strains, we are using that of the main type of persistence for which they are known for. As shown in our previous work, we acknowledge that persisters of both types can (and do) coexist under different conditions in not only the wild type E. coli strain but also on the DS1 and HipA7 strains. To diminish the influence of this complexity, we made sure to use a high dilution from (>1:30000) in all our exponential experiments to minimize the carryover of triggered persisters from the preceding stationary phase culture. As shown in Fig 6 and the supplementary photos from the protocol paper (PMID: 24586365), the isolated spontaneous persisters from the hipQ (DS1) strain exhibit the continuous and slow growth characteristic of their type, and < 5% of the spotted aliquot after persister isolation are yet to resume growth after 2 hours indicating a potentially low contamination with potential VBNCs even in stationary phase.

Overall, while we agree that the isolated persister population is likely heterogeneous, in the context of DS1/hipQ with approximately 100-fold higher spontaneous persister frequency, available data suggests the isolates are overwhelmingly dominated by spontaneous persisters, just as intended.

2. It is surprising that the genomic analysis is restricted to SNPs. Whole genome sequencing, particularly with the Illumina HiSeq platform and the analysis tools described, is also capable of identifying small insertions/deletions and in some cases larger chromosomal rearrangements or structural abnormalities. The authors do not explain why these classes of variation were not analyzed or reported. They should clarify whether such variants were detected and, if so, provide a rationale for why they were excluded from the study. If they did not identify them, then they should explain the limitations of their pipeline.

We apologize for the confusion. The analysis we performed included detection of small indels (insertions/deletions), as well as the whole assembly and annotation of the genome of the DS1 strain. We have modified the text to make it clearer. Whole genome alignment performed using progressiveMauve between the DS1 and KLY genomes also failed to reveal any indications of genome rearrangements or large-scale structural changes.

3. The authors compare exponential-phase DS1 cultures without treatment to the subpopulation of cells that survived their previously described chemical lysis protocol. I find this approach problematic. The chemical treatment does not necessarily select for bona fide persisters; rather, it may enrich for cells with altered membranes that resist lysis, or it may artificially induce dormancy and thereby confer antibiotic tolerance. The RNA-seq results presented by the authors in fact suggest that this possibility cannot be excluded. A stronger and more convincing approach would have been to directly treat exponential-phase cultures with ampicillin and perform transcriptomic analysis on the surviving unlysed cells. In doing so, the authors should avoid prolonged overnight cultures, as these can increase the frequency of VBNC cells. If exponential-phase cultures are free of VBNC, then antibiotic treatment should mainly enrich for persisters. This can be validated by quantifying survivors with flow cytometry and comparing those results with CFU measurements; matching values would confirm the persister enrichment, whereas discrepancies would indicate VBNC contamination. In addition, the possibility that VBNC cells are being isolated by the lysis method should be explicitly addressed, and the authors should provide verification that VBNC populations are not confounding their transcriptomic analyses.

We understand the concerns raised by the reviewer regarding the nature of the isolated cells and their identity as persisters. As previously published, the protocol used isolates cells that phenotypically exhibit the characteristics of each persister type (as shown by microscopy) in addition to corresponding to a similar fraction of the population as of that of the antibiotic isolated persisters published by other studies.

For example, in the previous published methods paper, when isolating spontaneous persisters in DS1/hipQ strain, >95% of the isolated cells exhibited slow growth with division rates characteristic of the DS1 spontaneous persisters. As a comparison, we also isolated persisters in the hipA7 strain, which is known to contain an elevated fraction of triggered persisters with extended dormancy before resuming regular, fast growth. In the hipA7 isolates, none of the enriched cells displayed the uniformly slow growth as was shown after isolating persisters in DS1/hipQ. But we did observe isolated hipA7 cells that suddenly resumed fast growth after extended dormancy (as expected for triggered persisters), which were not present for DS1/hipQ post-enrichment. To summarize these observations, available evidence suggests that the protocol most likely works as intended, it is unlikely that the protocol isolated something else that also happen to have the identical characteristics for both persister types.

Since the choice of our protocol raised so many questions, we decided to include some microscopic images in the supplementary section for explanatory purposes.

Although we would agree with the proposed post-treatment with ampicillin after isolation, the length of the antibiotic treatment allows for persisters to stocastically exit the persister state, significantly reducing the already limiting amount of persisters that could be analyzed. Further, as antibiotics are known to induce stress response, especially with such a lengthy treatment period, we worried it might generate further confounding factors.

In terms of preventing the accumulation of VBNCs, as noted before, we not only used a high dilution ratio for our exponential cultures, but also only used short stationary (<16 hours) cultures as started cultures. Although it is highly possible that a small fraction of VBNCs are present in the enriched samples of spontaneous persisters, as shown in the microscopy images of the protocol article (Fig 6 and SI) this fraction is likely negligible (< 5%).

4. The study does not go beyond descriptive transcriptomics. The authors did not perform any genetic manipulations (such as deletion of downregulated genes or overexpression of upregulated genes) to test whether these changes causally influence persistence. Such validation would be essential to move from correlation to mechanism.

This is correct, this study’s scope has aimed to help elucidate potential contributing mechanisms to spontaneous persisters as well as to help narrow down which mutations could be responsible for the hipQ phenotype of DS1. Further testing on a mechanistic cause is the subject of our next study.

5. The lipidomics study design is problematic because the authors use different strains and growth conditions, which makes direct comparisons difficult to interpret. According to their methods, lipids were extracted from E. coli MG1655 and DS1 during both exponential and stationary phase, while “triggered” and “spontaneous” persister cells were isolated from a stationary-phase culture of E. coli TH1269 and an exponential-phase culture of E. coli DS1, respectively. These conditions are not equivalent and introduce multiple variables, such as strain background, growth phase, and isolation protocol, that complicate the attribution of lipidomic differences specifically to persister physiology. I would have expected the authors to use consistent conditions and treatments across all three strains to allow for clearer and more meaningful comparisons.

We thank the reviewer for this comment and apologize for the confusion. We have now removed the lipidomic results relating to the triggered persisters, which we understand now were generating confusion. However, we would like to explain our rationale for the experimental design used. Our design was focused on separately observing the changes in each persister type compared to the strain from which they were generated both in exponential and in stationary phase. For example, for DS1 spontaneous persisters that are generated in exponential phase, we compared their lipid profiles to those of DS1 exponentially growing cells and DS1 stationary phase cells. Similarly, for the triggered persisters, we isolated persisters from MG1655 hipA7 (TH1269) in stationary phase and compared those profiles to those of MG1655 in exponential and stationary phase. As TH1629 only differs from MG1655 in a point mutation on the HipA protein, we used the MG1655 background more cleanly to observe the stationary phase profiles, given the high frequency of triggered persisters present on these conditions.

Our intent was to observe the normal range of change in the fatty acid profiles between exponential and stationary phase for each strain separately and noted how the change was more drastic when comparing each persister state to their respective “background”. However, as this seems to generate confusion, we have removed the lipidomic analysis of triggered persisters from the manuscript.

Attachment Submitted filename: Rebuttal_PloSOne_20260115.docx

# Peer review: Decision Letter 1

**DOI:** 10.1371/journal.pone.0351161.r003

**Authors:** Bashir Sajo Mienda

16 Mar 2026

<div>PONE-D-25-43801R1

An integrative multi-omics approach points to membrane composition as a key factor in E. coli persistence

PLOS One

Dear Dr. Canas-Duarte,

Thank you for submitting your manuscript to PLOS ONE. After careful consideration, we feel that it has merit but does not fully meet PLOS ONE’s publication criteria as it currently stands. Therefore, we invite you to submit a revised version of the manuscript that addresses the points raised during the review process.

Please submit your revised manuscript by Apr 30 2026 11:59PM. If you will need more time than this to complete your revisions, please reply to this message or contact the journal office at plosone@plos.org. When you're ready to submit your revision, log on to https://www.editorialmanager.com/pone/ and select the 'Submissions Needing Revision' folder to locate your manuscript file.

Please include the following items when submitting your revised manuscript:

- A letter that responds to each point raised by the academic editor and reviewer(s). You should upload this letter as a separate file labeled 'Response to Reviewers'.
- A marked-up copy of your manuscript that highlights changes made to the original version. You should upload this as a separate file labeled 'Revised Manuscript with Track Changes'.
- An unmarked version of your revised paper without tracked changes. You should upload this as a separate file labeled 'Manuscript'.

-->If you would like to make changes to your financial disclosure, please include your updated statement in your cover letter. Guidelines for resubmitting your figure files are available below the reviewer comments at the end of this letter.

If applicable, we recommend that you deposit your laboratory protocols in protocols.io to enhance the reproducibility of your results. Protocols.io assigns your protocol its own identifier (DOI) so that it can be cited independently in the future. For instructions see: https://journals.plos.org/plosone/s/submission-guidelines#loc-laboratory-protocols. Additionally, PLOS ONE offers an option for publishing peer-reviewed Lab Protocol articles, which describe protocols hosted on protocols.io. Read more information on sharing protocols at https://plos.org/protocols?utm_medium=editorial-email&utm_source=authorletters&utm_campaign=protocols.

We look forward to receiving your revised manuscript.

Kind regards,

Bashir Sajo Mienda, PhD

Academic Editor

PLOS One

**Journal Requirements:**

1. If the reviewer comments include a recommendation to cite specific previously published works, please review and evaluate these publications to determine whether they are relevant and should be cited. There is no requirement to cite these works unless the editor has indicated otherwise.

2. Please review your reference list to ensure that it is complete and correct. If you have cited papers that have been retracted, please include the rationale for doing so in the manuscript text, or remove these references and replace them with relevant current references. Any changes to the reference list should be mentioned in the rebuttal letter that accompanies your revised manuscript. If you need to cite a retracted article, indicate the article’s retracted status in the References list and also include a citation and full reference for the retraction notice.

[Note: HTML markup is below. Please do not edit.]

Reviewers' comments:

Reviewer's Responses to Questions

**Comments to the Author**

1. If the authors have adequately addressed your comments raised in a previous round of review and you feel that this manuscript is now acceptable for publication, you may indicate that here to bypass the “Comments to the Author” section, enter your conflict of interest statement in the “Confidential to Editor” section, and submit your "Accept" recommendation.

Reviewer #1: (No Response)

**********

2. Is the manuscript technically sound, and do the data support the conclusions?

The manuscript must describe a technically sound piece of scientific research with data that supports the conclusions. Experiments must have been conducted rigorously, with appropriate controls, replication, and sample sizes. The conclusions must be drawn appropriately based on the data presented.

Reviewer #1: Yes

**********

3. Has the statistical analysis been performed appropriately and rigorously?

Reviewer #1: Yes

**********

4. Have the authors made all data underlying the findings in their manuscript fully available?

The PLOS Data policy requires authors to make all data underlying the findings described in their manuscript fully available without restriction, with rare exception (please refer to the Data Availability Statement in the manuscript PDF file). The data should be provided as part of the manuscript or its supporting information, or deposited to a public repository. For example, in addition to summary statistics, the data points behind means, medians and variance measures should be available. If there are restrictions on publicly sharing data—e.g. participant privacy or use of data from a third party—those must be specified.

Reviewer #1: (No Response)

**********

5. Is the manuscript presented in an intelligible fashion and written in standard English?

PLOS ONE does not copyedit accepted manuscripts, so the language in submitted articles must be clear, correct, and unambiguous. Any typographical or grammatical errors should be corrected at revision, so please note any specific errors here.

Reviewer #1: Yes

**********

6. Review Comments to the Author

Please use the space provided to explain your answers to the questions above. You may also include additional comments for the author, including concerns about dual publication, research ethics, or publication ethics. (Please upload your review as an attachment if it exceeds 20,000 characters)

Reviewer #1: While I appreciate the authors’ detailed responses, several concerns remain at the level of framing and language rather than experimental design (I am not requesting additional experiments at this point). However, I strongly recommend further editing of the manuscript text to better align conclusions with what is directly supported by the data:

1) The manuscript continues to rely on a strict “spontaneous vs triggered” classification and frequently refers to the isolated DS1 population as spontaneous persisters. Given the heterogeneity acknowledged by the authors and the nature of the isolation protocol, the text should consistently use more descriptive language such as “persisters isolated from DS1 during exponential phase” rather than categorical labels, including in the Abstract and Discussion.

2) Statements implying that spontaneous persisters are uniformly slow-growing should be corrected. Spontaneous persisters do not have to be slow-growing; they can also be non-growing, and cells can stochastically enter non-growing states during exponential growth. The text as well as Figure 1 should reflect this broader and more established view of persister physiology.

3) The manuscript repeatedly implies a causal or mechanistic role for membrane composition in persister formation, although the data support only association. The text should be revised throughout to remove causal/mechanistic implications and instead describe membrane changes as correlated with or associated with persistence.

4) The Discussion should explicitly acknowledge that the chemical lysis-based enrichment could preferentially select for cells with altered membrane properties or other non-persister cells.

**********

7. PLOS authors have the option to publish the peer review history of their article (what does this mean?). If published, this will include your full peer review and any attached files.

If you choose “no”, your identity will remain anonymous but your review may still be made public.

**Do you want your identity to be public for this peer review?**  For information about this choice, including consent withdrawal, please see our Privacy Policy.

Reviewer #1: No

**********

[NOTE: If reviewer comments were submitted as an attachment file, they will be attached to this email and accessible via the submission site. Please log into your account, locate the manuscript record, and check for the action link "View Attachments". If this link does not appear, there are no attachment files.]

To ensure your figures meet our technical requirements, please review our figure guidelines: https://journals.plos.org/plosone/s/figures

You may also use PLOS’s free figure tool, NAAS, to help you prepare publication quality figures: https://journals.plos.org/plosone/s/figures#loc-tools-for-figure-preparation.

NAAS will assess whether your figures meet our technical requirements by comparing each figure against our figure specifications.

# Author comment: Author response to Decision Letter 2

**DOI:** 10.1371/journal.pone.0351161.r004

29 Apr 2026

1) The manuscript continues to rely on a strict “spontaneous vs triggered” classification and frequently refers to the isolated DS1 population as spontaneous persisters. Given the heterogeneity acknowledged by the authors and the nature of the isolation protocol, the text should consistently use more descriptive language such as “persisters isolated from DS1 during exponential phase” rather than categorical labels, including in the Abstract and Discussion.

Response: We thank the reviewer for their comments and thoughtful consideration of our study. We appreciate the suggestion for more careful wording throughout the text, and we have used the recommended description where appropriate.

We understand that the concern here is that although DS1 is widely acknowledged as a model organism for spontaneous persistence, the isolated population after the enrichment protocol is not guaranteed to be 100% pure. To address this, the use of “spontaneous persisters” is now largely limited to conceptual context. We have also included an explicit description of the precautions taken to minimize the fraction of non-spontaneous persisters in the isolated samples analyzed.

2) Statements implying that spontaneous persisters are uniformly slow-growing should be corrected. Spontaneous persisters do not have to be slow-growing; they can also be non-growing, and cells can stochastically enter non-growing states during exponential growth. The text as well as Figure 1 should reflect this broader and more established view of persister physiology.

Response: We thank the reviewer for this insightful suggestion. We agree that the growth dynamics (or lack thereof) of spontaneous persisters may not be universally slow-growing outside of the E. coli strains for which this growth phenotype has been described. As such, we have revised the text and the Figure 1 legend accordingly to constrain this description to the DS1 strain.

We would like, however, to provide some context for our original intention. The characterization of spontaneous (Type II) persisters as slow-growing is based on the seminal work by Balaban and coauthors in 2004 with the DS1 (hipQ) strain, the canonical model in which this persister type was discovered and observed to continue growing and dividing slowly during antibiotic treatment (PMIDs: 15308767, 24586365). We recognize, however, that the growth behavior of spontaneous persisters in other strains or species has simply not been investigated in detail. This slow-growth phenotype may be specific to the few E. coli strains for which this type of data has been reported. The 2019 joint review by Balaban et al. (PMID: 30980069), which represents the current consensus definition of persister types, notably does not specify any particular growth trait for spontaneous persisters. Interestingly, we recently had a chat with Prof. Dan Andersson, a co-author of that review. He was surprised to hear that spontaneous persisters in DS1/hipQ actually exhibit growth. This is a strong sign that this nuance is not widely appreciated in the field.

In the revised manuscript, we have therefore kept the description that spontaneous persisters in DS1 are slow-growing, but also carefully rephrased the general description to be open to the possibility that spontaneous persisters in other strains or species could display different growth characteristics, including non-growth. We hope this now accurately reflects both the original observations and the broader perspective raised by the reviewer.

3) The manuscript repeatedly implies a causal or mechanistic role for membrane composition in persister formation, although the data support only association. The text should be revised throughout to remove causal/mechanistic implications and instead describe membrane changes as correlated with or associated with persistence.

Response: We thank the reviewer for this comment and have modified the text accordingly. Specifically, we have changed the language referring to the potential role of the membrane in persistence to indicate correlation and involvement rather than causality.

4) The Discussion should explicitly acknowledge that the chemical lysis-based enrichment could preferentially select for cells with altered membrane properties or other non-persister cells.

Response: We thank the reviewer for this suggestion and have now added this acknowledgement in the discussion.

Attachment Submitted filename: ResponseToReviewers_20260425.docx

# Peer review: Decision Letter 2

**DOI:** 10.1371/journal.pone.0351161.r005

**Authors:** Bashir Sajo Mienda

24 May 2026

An integrative multi-omics approach points to membrane composition as a key factor in E. coli persistence

PONE-D-25-43801R2

Dear Dr. CANAS-DUARTE,

We’re pleased to inform you that your manuscript has been judged scientifically suitable for publication and will be formally accepted for publication once it meets all outstanding technical requirements.

Within one week, you’ll receive an e-mail detailing the required amendments. When these have been addressed, you’ll receive a formal acceptance letter and your manuscript will be scheduled for publication.

An invoice will be generated when your article is formally accepted. Please note, if your institution has a publishing partnership with PLOS and your article meets the relevant criteria, all or part of your publication costs will be covered. Please make sure your user information is up-to-date by logging into Editorial Manager at Editorial Manager® and clicking the ‘Update My Information' link at the top of the page. For questions related to billing, please contact billing support.

If your institution or institutions have a press office, please notify them about your upcoming paper to help maximize its impact. If they’ll be preparing press materials, please inform our press team as soon as possible -- no later than 48 hours after receiving the formal acceptance. Your manuscript will remain under strict press embargo until 2 pm Eastern Time on the date of publication. For more information, please contact onepress@plos.org.

Kind regards,

Bashir Sajo Mienda, PhD

Academic Editor

PLOS One

Additional Editor Comments (optional):

Reviewers' comments:

Reviewer's Responses to Questions

**Comments to the Author**

1. If the authors have adequately addressed your comments raised in a previous round of review and you feel that this manuscript is now acceptable for publication, you may indicate that here to bypass the “Comments to the Author” section, enter your conflict of interest statement in the “Confidential to Editor” section, and submit your "Accept" recommendation.

Reviewer #2: All comments have been addressed

**********

2. Is the manuscript technically sound, and do the data support the conclusions?

The manuscript must describe a technically sound piece of scientific research with data that supports the conclusions. Experiments must have been conducted rigorously, with appropriate controls, replication, and sample sizes. The conclusions must be drawn appropriately based on the data presented.

Reviewer #2: Yes

**********

3. Has the statistical analysis been performed appropriately and rigorously?

Reviewer #2: Yes

**********

4. Have the authors made all data underlying the findings in their manuscript fully available?

The PLOS Data policy requires authors to make all data underlying the findings described in their manuscript fully available without restriction, with rare exception (please refer to the Data Availability Statement in the manuscript PDF file). The data should be provided as part of the manuscript or its supporting information, or deposited to a public repository. For example, in addition to summary statistics, the data points behind means, medians and variance measures should be available. If there are restrictions on publicly sharing data—e.g. participant privacy or use of data from a third party—those must be specified.

Reviewer #2: Yes

**********

5. Is the manuscript presented in an intelligible fashion and written in standard English?

PLOS ONE does not copyedit accepted manuscripts, so the language in submitted articles must be clear, correct, and unambiguous. Any typographical or grammatical errors should be corrected at revision, so please note any specific errors here.

Reviewer #2: Yes

**********

6. Review Comments to the Author

Please use the space provided to explain your answers to the questions above. You may also include additional comments for the author, including concerns about dual publication, research ethics, or publication ethics. (Please upload your review as an attachment if it exceeds 20,000 characters)

Reviewer #2: (No Response)

**********

7. PLOS authors have the option to publish the peer review history of their article (what does this mean?). If published, this will include your full peer review and any attached files.

If you choose “no”, your identity will remain anonymous but your review may still be made public.

**Do you want your identity to be public for this peer review?**  For information about this choice, including consent withdrawal, please see our Privacy Policy.

Reviewer #2: No

**********

# Editor report: Acceptance letter

**DOI:** 10.1371/journal.pone.0351161.r006

**Authors:** Bashir Sajo Mienda

PONE-D-25-43801R2

PLOS One

Dear Dr. Canas-Duarte,

I'm pleased to inform you that your manuscript has been deemed suitable for publication in PLOS One. Congratulations! Your manuscript is now being handed over to our production team.

At this stage, our production department will prepare your paper for publication. This includes ensuring the following:

* All references, tables, and figures are properly cited

* All relevant supporting information is included in the manuscript submission,

* There are no issues that prevent the paper from being properly typeset

You will receive further instructions from the production team, including instructions on how to review your proof when it is ready. Please keep in mind that we are working through a large volume of accepted articles, so please give us a few days to review your paper and let you know the next and final steps.

Lastly, if your institution or institutions have a press office, please let them know about your upcoming paper now to help maximize its impact. If they'll be preparing press materials, please inform our press team within the next 48 hours. Your manuscript will remain under strict press embargo until 2 pm Eastern Time on the date of publication. For more information, please contact onepress@plos.org.

You will receive an invoice from PLOS for your publication fee after your manuscript has reached the completed accept phase. If you receive an email requesting payment before acceptance or for any other service, this may be a phishing scheme. Learn how to identify phishing emails and protect your accounts at https://explore.plos.org/phishing.

If we can help with anything else, please email us at customercare@plos.org.

Thank you for submitting your work to PLOS ONE and supporting open access.

Kind regards,

PLOS ONE Editorial Office Staff

on behalf of

Dr. Bashir Sajo Mienda

Academic Editor

PLOS One
